# The Assessment of Water Retention Efficiency of Different Soil Amendments in Comparison to Water Absorbing Geocomposite

**DOI:** 10.3390/ma14216658

**Published:** 2021-11-04

**Authors:** Michał Śpitalniak, Adam Bogacz, Zofia Zięba

**Affiliations:** 1 Institute of Environmental Engineering, The Faculty of Environmental Engineering and Geodesy, Wrocław University of Environmental and Life Sciences, Grunwaldzki Sq. 24, 50-363 Wroclaw, Poland; 2 Institute of Soil Sciences and Environmental Protection, The Faculty of Life Sciences and Technology, Wrocław University of Environmental and Life Sciences, ul. Grunwaldzka 53, 50-357 Wroclaw, Poland; adam.bogacz@upwr.edu.pl; 3 Department of Civil Engineering, The Faculty of Environmental Engineering and Geodesy, Wrocław University of Environmental and Life Sciences, Grunwaldzki Sq. 24, 50-363 Wroclaw, Poland; zofia.zieba@upwr.edu.pl

**Keywords:** soil amendment, soil additive, water retention, water absorbing geocomposite, soil moisture, loamy sand

## Abstract

Soil amendments are substances added to the soil for moisture increment or physicochemical soil process enhancement. This study aimed to assess the water conservation efficiency of available organic soil amendments like bentonite, attapulgite, biochar and inorganics like superabsorbent polymer, and nonwoven geotextile in relation to the newly developed water absorbing geocomposite (WAG) and its biodegradable version (bioWAG). Soil amendments were mixed with loamy sand soil, placed in 7.5 dm^3^ pots, then watered and dried in controlled laboratory conditions during 22-day long drying cycles (pot experiment). Soil moisture was recorded in three locations, and matric potential was recorded in one location during the drying process. The conducted research has confirmed that the addition of any examined soil amendment in the amount of 0.7% increased soil moisture, compared to control, depending on measurement depth in the soil profile and evaporation stage. The application of WAG as a soil amendment resulted in higher soil moisture in the centre and bottom layers, by 5.4 percent point (p.p.) and 6.4 p.p. on day 4 and by 4.5 p.p. and 8.8 p.p. on day 7, respectively, relative to the control samples. Additionally, an experiment in a pressure plate extractor was conducted to ensure the reliability of the obtained results. Soil density and porosity were also recorded. Samples containing WAG had water holding capacity at a value of −10 kPa higher than samples with biochar, attapulgite, bentonite, bioWAG and control by 3.6, 2.1, 5.7, 1 and 4.5 percentage points, respectively. Only samples containing superabsorbent polymers and samples with nonwoven geotextiles had water holding capacity at a value of −10 kPa higher than WAG, by 14.3 and 0.1 percentage points, respectively. Significant changes were noted in samples amended with superabsorbent polymers resulting in a 90% soil sample porosity and bulk density decrease from 1.70 g∙cm^−3^ to 1.14 g∙cm^−3^. It was thus concluded that the water absorbing geocomposite is an advanced and most efficient solution for water retention in soil.

## 1. Introduction

Soil amendments used to increase soil water retention capacity can be classified into two groups, based on their origin. One of the groups includes soil amendments that are formed as a result of natural processes, such as attapulgite, bentonite, zeolite or unprocessed wood waste. The second group is that of soil amendments of anthropogenic origin. This group will include amendments like biochar, superabsorbent polymer (SAP), nonwoven geotextiles, water absorbing geocomposite (WAG) or biodegradable water absorbing geocomposite (bioWAG). Their function is to retain additional amounts of water in the soil profile through a reduction of water infiltration or evaporation [1,2,3,4,5,6]. Water conservation is essential due to the high level of water consumption for agricultural purposes and progressing climate change. As much as 70–80% of fresh water in the world is used for agricultural purposes [7,8,9,10]. Predictions related to mean world ambient temperature increases and the soil moisture index by Grillakis [11] indicate the necessity of developing new solutions for coping with water deficits or effects of droughts [12,13,14,15,16,17].

Water retention in soil by soil amendments is an extensive subject matter. The mechanism of water retention in unsaturated soil is based on capillary forces and on the sorptive potential of more complex soil particles, where the soil sorptive potential depends on physicochemical potentials of an electromagnetic nature, like van der Waals intermolecular interactions, exchangeable cations, surface hydroxyls or an electrical double layer [18,19,20,21,22,23]. The influence of exchangeable cations on water absorbency in soils was investigated, e.g., by Dontsova et al. [19], Woodruff and Revil [24]. The drying–wetting history of soil is also significant [25,26]. Therefore, if we modify the soil by adding soil amendments, we will additionally analyse the effect of parameters characteristic of the amendment applied on water retention in the soil. The parameters described most frequently in the literature are the porosity of the material used, cation exchange capacity, open surface area, or porosity, making those materials considered sorptive [27,28,29,30].

One of the more straightforward methods of improving the water conditions of sandy soil is the addition of clay soil or clay minerals. They retain water due to their unusual crystal structure and high specific surface area, which induces their specific physicochemical properties, such as porosity or dehydration [27]. Considering bentonite, one should also take into account its swelling capacity. The application of bentonite as a soil treatment results in higher water efficiency use and a decrease of drought stress [1,31,32,33]. In sandy soil, bentonite can reduce both the capillary rise and water infiltration, the effect of which can be the desired moisture control [31]. Numerous studies describe the possibility of using attapulgite in agriculture as an agent supporting plant growth or increasing soil moisture [2,34,35,36].

With the development of technology and knowledge, the usage of various mixes of amendments and improvements of their production processes, intending to enhance the water retention capacity of soil have been observed. In recent years, biochar has enjoyed much interest. Biochar is formed as a result of the pyrolysis of organic matter. According to the research, a higher temperature of pyrolysis leads to an increase of specific surface area, which subsequently results in increased levels of water absorption in soil [28,37]. Along with pyrolysis temperature, the kind of feedstock from which biochar is produced is also essential [38], as it determines its hydrophobicity [39]. However, biochar does not only offer possibility of water retention, as an impact on the formation of soil aggregates has been demonstrated [40,41,42,43], has has its roles in the modification of soil structure and hydraulic conductivity [44,45,46,47,48]. Even the form of the amendment applied, by analogy to soil particles, affects water movement in soil [49]. The change of the physicochemical properties of biochar in soil over time is another process not always exerting a positive effect on the maintenance of moisture in the soil [50]. Therefore, water retention by biochar is strongly dependent on soil and biochar properties [51,52,53,54], which can account for the variation in the available research results. Numerous critical studies presented in the area of biochar utilisation suggest a lack of evidence for improvements in crop water availability and, therefore, crop yields [55,56]. Some authors question the capacity of biochar to absorb water, especially in sandy soils [57], while others indicate an adverse modification of physicochemical parameters of soil [32]. Depending on the soil type, the amount of biochar required to achieve satisfactory results in soil moisture is often relatively significant and somewhat challenging to apply in practice [58,59]. However, in most studies, biochar is presented favourably as an amendment that fulfils its function well [60,61,62,63,64,65,66].

The water retention capacity of most soil amendments is small compared to superabsorbent polymers (SAP) that retain large amounts of water and transform into a gel. SAPs are synthetic, three-dimensional, cross-linked polymers. One gram of SAP can absorb up to 500 g of distilled water [67,68,69,70]. According to Laftah et al. [71], up to 1000 g of water can be retained per 1 g of SAP. However, the presence of ions or salts reduces the capacity of superabsorbent polymers to absorb water [72,73]. Depending on the conditions, that process can be dynamic or intensify over time, and therefore it is referred to as ageing. In a study conducted by Banedjschafie and Durner [74], six months after application to soil (and after numerous swelling-drying cycles), SAP lost its capacity to retain water in the soil to a significant degree.

Another factor that determines the water absorption capacity of SAP to a given degree is free space. By absorbing water, SAP swells, increasing its volume. Because SAP is usually used by mixing it with soil, the depth of application and the density of the soil are vital parameters, reflected in the soil load that has to be overcome so that SAP can swell effectively and thus retain water in the soil in the form of a hydrogel. Research in this area was conducted by Lejcuś et al. [75] and Misiewicz et al. [76,77]. Differences in the quantity of water retained between freely swelling samples and samples swelling under a load reached up to 94% [75]. In relation to the swelling pressure generated [77], certain types of SAP have a significant impact on soil bulk density and porosity, the effects of which have been described by Han et al. [78] and Bai et al. [79]. In the case of SAP application in relatively large amounts, changes can be observed in the values of soil shear strength or hydraulic conductivity [80]. A positive effect of SAP in the context of soil moisture or increase of yields has been noted by numerous authors [72,81,82,83,84,85,86].

Efforts aimed at optimum utilisation of the extraordinary properties of superabsorbent polymers resulted in the creation of water absorbing geocomposite (WAG) [87]. WAG is a 3D structure designed to retain water at any desired depth in the soil profile. Depending on the planned application, it can be used in the form of a mat or in a linear or point-wise manner. WAG in the form of a mat is built of a synthetic openwork spatial mesh that supports the load of soil. The structure is wrapped with a suitable geotextile with hydrophilic properties, while the interior is filled with a superabsorbent polymer. Water is sucked into the WAG structure interior through the geotextile. Then, SAP retains the water in the form of gel, and the spatial openwork construction ensures space for further SAP operation. As a result of such a design, SAP can change its volume many times in the course of the swelling or relaxation processes [6]. Ultimately, up to 95% of the retained water is available for plants when plant roots penetrate the WAG structure [88,89]. In the other types of WAG (point and linear), the principle of operation is the same, only the shape differs.

The influence of WAG in the form of a ma, placed in the soil profile on soil moisture and matric potential, was the subject of a study by Śpitalniak et al. [90]. The conducted research demonstrated that WAG in the form of a mat permitted the retention of up to six-fold more water than the control samples [90]. In another study, it was confirmed that the application of WAG in the soil profile resulted in an increase of grass root volume by 130%, root length by up to 29%, mass of the overground parts of plants by 83%, and shearing strength of the layer of soil overgrown by roots by 20% for grass and 66% for shrubs [6]. The effect of WAG on plants and the physicochemical properties of soil was also described in studies by Bąbelewski et al. [91], Cabała et al. [92] and Pancerz et al. [93]. In a study by Wróblewska et al. [94], the application of WAG to soil resulted in an increase of *Brunnera macrophylla* biomass by up to 360% and the number of leaves by 60%. WAG has also been described as useful in typical engineering applications, in biotechnological structures or in the remediation of degraded areas [5,95,96,97]. Moreover, a biodegradable version which differs in weaving type and skeleton material is under development [98].

Nonwoven geotextile can also be applied to soil on its own. It is used primarily for the separation of soil layers or drainage to modify moisture in the soil profile through moisture movement control. When encountering a barrier in the form of a geosynthetic layer, water infiltrating an unsaturated clay soil can increase its water storage [99,100] up to 0.3–0.5 m above this layer at a low infiltration rate [101]. In such a situation, soil moisture can increase up to 46% (soil saturation of 0.93) above the geosynthetic layer before free drainage through the geosynthetic layer occurs, and at the height of 0.5 m above the geosynthetic layer soil moisture can increase up to 24% [102]. Most frequently, at a pressure lower than −1 kPa on the interface between the soil and the geosynthetic layer, water movement through the geosynthetic barrier ceases [101,103]. The passage of water becomes possible again when the soil layer above the geosynthetic layer attains full saturation when a suitable hydraulic gradient is achieved, until finally a breakthrough phenomenon occurs [104]. Although the literature does not provide any exhaustive description of the application of geotextiles in the soil profile for moisture conservation, there are some reports on the application of geotextiles on soil surface to reduce evaporation [105,106]. Synthetic geotextiles with wicking properties, which can drain the soil even when it is unsaturated are also of interest [107,108]. Due to their three-dimensional structures, i.e., specific weaving patterns of fibres and pore size diameter, synthetic geotextilesacquire the ability of water suction through fibre channels [107,109]. This is also the case of the nonwoven geotextile used in WAG.

The benefits of WAG usage in terms of environmental impact are known and have been described in the works referenced above. However, WAG efficiency has never been benchmarked to other widely known and available soil amendments. Therefore, the objective of this study presented herein was to verify the retention capacity of WAG compared to other soil amendments in identical operating conditions. The published research on soil amendments often lacks the essential parameters necessary to compare the efficiency of soil amendments. Providing a reliable comparison without conducting an experiment would thus be challenging, as multiple factors have to be considered in this type of research, including soil type and texture, the density and moisture of the soil, the temperature and duration of the evaporation process, the amount of soil amendment used, the amount of water used for the experiment, and, finally, the placement of the amendment in the soil. Therefore, a pot experiment and an additional experiment involving a pressure plate extractor were planned to answer the above research question. To fulfil this objective, the WAG was compared under the same operating conditions with both natural (bentonite, attapulgite) and anthropogenic soil amendments (biochar, SAP, bioWAG and nonwoven geotextile).

## 2. Materials and Methods

The study was conducted in the laboratories of the Wrocław University of Environmental and Life Sciences. Seven soil amendments were selected for the analyses: attapulgite, superabsorbent polymer, biochar, bentonite, WAG, bioWAG and nonwoven geotextile. They were mixed with soil or placed in the samples as a layer. In the pot experiment, the samples were watered, and the process of their drying in controlled temperature conditions was observed.

### 2.1. Preparation of Pot Samples

For the purposes of the experiment, PVC pots with a volume of 7.5 L were used. The perforated bottom of the pots was lined with a draining mat and geotextile to enable draining excess water. The soil used in the experiment was a loamy sand soil. Stones, roots and parts of plants were removed from the soil. The soil was crushed and screened through a sieve with 2.0 mm mesh to achieve soil particles with a fairly constant size distribution. Next, it was mixed and dried to obtain uniform soil moisture. The pots were filled with the soil, controlling its volume and mass so as to obtain a uniform soil density of 1.70 g∙cm^−3^ in the entire soil profile. As treatments, one of the soil amendments was applied in each of the pots. Each treatment was replicated three times. In addition, three control samples were created without any soil amendments. Therefore, a total of 24 pot samples were prepared.

**Figure 1:**
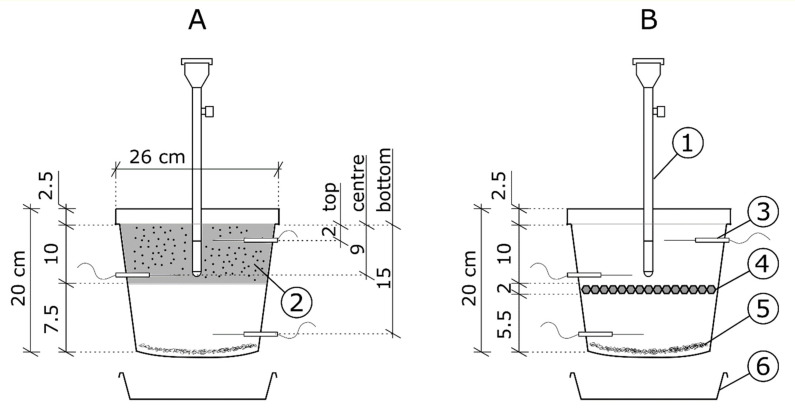
Scheme of sample preparation. (**A**) Pot samples with granular soil amendments (biochar, attapulgite, bentonite, SAP), and (**B**) pot samples with solid state soil amendments (WAG, bioWAG, geotextile). The numbers represent 1—probe for the measurement of soil matric potential, 2—a volume of soil with mixed granular amendment, 3—probe for the measurement of soil moisture, 4—a solid amendment applied as a layer, 5—a drainage mat with geotextile, 6—a tray for effluent. Line marks on the left side of pots (**A**,**B**) indicate distinct layers. Line marks on the right side of pot A indicate the positioning of the measuring probes in the profile.

Granular soil amendments were mixed with the soil before it was placed in the pots. To be exact, the granular soil amendments were mixed with a soil mass equivalent to the mass of the soil layer with a thickness of 10 cm, measuring from the upper rim of the pot, in the amount of 0.7% of the weight of that mass. The addition of 7.5 g of soil amendment per one kg of soil resulted in 44 g of soil amendment being placed in each pot. Converted for area, this was the equivalent of 8800 kg∙ha^−1^ of soil amendment. Solid soil amendments, such as WAG, bioWAG or geotextile, were placed at a depth of 10 cm in the course of filling the pots with soil. At the moment of application, the soil amendments used were dry. As the soil amendments used differed in the form of their application to soil, Figure 1 presents the structures of samples with granular and solid soil amendments. In spite of the differences in the methods of application or the forms of the amendments, the amounts were equivalent.

### 2.2. Testing Procedure in a Pot Experiment

The pot experiment was conducted in a laboratory provided with a thermal control system consisting of thermal insulation of the room, an air heater and air moisture absorber. The prepared samples were placed on tables in a random sequence, with taken care that samples containing the same amendment were not be placed next to one another to reduce the potential effect of zonal air circulation on the drying of the samples. Prior to installation in the pots, the measuring equipment was calibrated and used in a series of test measurements. After the preparation of the samples and the installation of the probes, the experiment was started. Each sample was watered with three litres of water. Water was poured onto a drainage mat placed on the surface of the pots to ensure uniform wetting of the samples. The process of watering each sample was extended in time and lasted for 35 min. After watering, the drainage mat was removed. The trays placed beneath the pots collected small amounts of water, not exceeding 3% of the total water dose used, they were therefore excluded from the subsequent analysis. Next, the drying of the samples at a constant temperature of approximately 30 °C was monitored for 24 days.

Moisture measurement was conducted in every pot at three depths, i.e., at 2 cm, 9 cm and 15 cm below soil surface. Each pot was equipped with a tensiometer for the measurement of soil matric potential, and they were installed such that the ceramic filter was positioned in the soil volume containing the soil amendment. The tensiometers were regularly vented during the course of measurements. The water solution in the tensiometers was a mixture of distilled water and the tensiometer liquid supplied by the manufacturer. In addition, TDR sensors were used to monitor the temperature at four points in the laboratory, at the height of the upper rim of the pots. The experiment was performed in three replicates.

### 2.3. Equipment

In the pot experiment, soil moisture was measured using the time domain reflectometry (TDR) technique, using the TDR/MUX/mpts device with LP/ms TDR probes manufactured by the company ETEST (Lublin, Poland). The use of this TDR apparatus has been described in publications by Janik et al. [110], Malicki and Skierucha [111], Skierucha [112] and Skierucha et al. [113]. Measurements of soil matric potential were performed with the use of liquid tensiometers made by the company Irrometer (Riverside, CA, USA), equipped with electronic pressure sensors and a data logger.

With the use of a ceramic pressure plate extractor, the soil water characteristics of the amended soils were determined. Four samples were taken from each of the pots. As a result, twelve samples from each treatment were examined. Therefore, in total, 120 samples were examined in the pressure plate extractor and sandbox. Undisturbed samples taken with 100 cm^−3^ steel metal rings were used to determine soil water potential at low pressure (<300 kPa) using the tensiometric sandbox technique [114]. Disturbed soil samples were used for soil analyses, and the determination of the relationship between soil water potential and soil water content at higher pressure (300–1550 kPa) was performed using ceramic pressure plates (5 bar and 15 bar) in a chamber apparatus [115] from the company Soil Moisture Equipment Corp. Samples for the analyses were taken from the top layer of soil in the pots, after the termination of the original experiment.

### 2.4. Materials Used

#### 2.4.1. Soil

The loamy sand soil used in the experiment was acquired from the area of the Swojec Agricultural Experimental Station located in Wrocław, Lower Silesia, Poland (51°06′53″ N, 17°08′27″ E), belonging to the Wrocław University of Environmental and Life Sciences. According to typological soil classification, alluvial soils that are typical of river valleys are dominant in the area of the Swojec Agricultural Experimental Station [116]. In the international classification FAO-WRB (IUSS 2015), theis soil is classified in the reference group of Arenosols (Eutric Fluvic Brunic Arenosols (Aric))[117]. One bulk soil sample from the surface (0–30 cm) was collected.

#### 2.4.2. Attapulgite and Granular Bentonite

The attapulgite used in the experiment consisted of colloidal magnesium and aluminium hydrosilicate mined in the vicinity of Attapulgus, Georgia (USA). Attapulgite is a thermally activated, powdered attapulgite mineral that has a high level of sorptivity. The chemical composition of attapulgite can be expressed with the formula (Mg,Al)_5_Si_8_O_20_·4H_2_O. It is characterised by the form of pulverised powder.

The bentonite used in the experiment is a natural silicate mineral containing montmorillonite with a lamellar structure. Its properties include water retention properties and swelling ability. Granulated bentonite is characterised by grey colouring and granularity in the range of 2–5 mm. In the experiment, it was used in a granular form. Analysis of the chemical composition of the amendments was performed with the method of X-ray diffraction (XRD) for the needs of this study (Table S1).

#### 2.4.3. Nonwoven Geotextile

Nonwoven Geotextile made in 100% of Polypropylene, UV stabilised, with a grammage of 90 g∙m^−2^ was used in the experiment. The characteristic opening size (O_90_), determined on the basis of the standard EN ISO 12956, was 175 µm. The water flow rate at 10 cm water height, acc. to standard BS 6906-3, was 175 dm^3^∙m^−2^∙s^−1^. The data were acquired from the product data sheet of the Dupont Typar SF geotextile. It is important to note that this particular nonwoven geotextile does not have wicking abilities.

#### 2.4.4. Water Absorbing Geocomposite (WAG)

**Figure 2:**
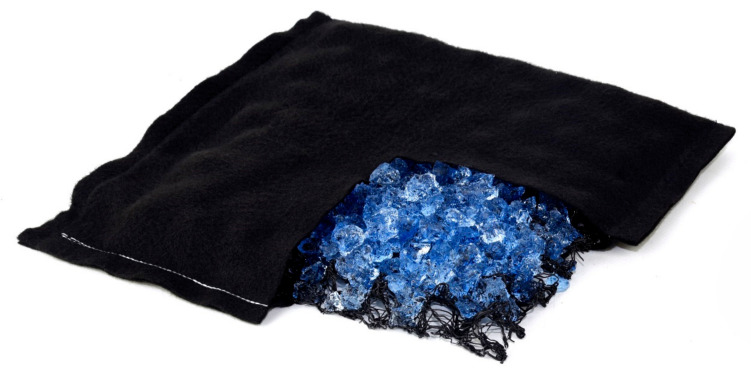
Cross-section of water absorbing geocomposite. Visible nonwoven geotextile, spatial openwork structure and superabsorbent polymer in a saturated state.

A water absorbing geocomposite (WAG) in the form of a mat was used in the pot samples as a treatment (Figure 2). The WAG consisted of a nonwoven geotextile, synthetic openwork spatial mesh as skeleton structure and a superabsorbent polymer placed inside the structure. A polyester needle-punched nonwoven geotextile made of 100% polyester with high wicking abilities and a grammage of 150 g∙m^−2^ was used. Superabsorbent polymer, Aquasorb 3005 KL (SNF Floerger, Andrézieux, France), a cross-linked copolymer of acrylamide and potassium acrylate, was used as a water retainer in the WAG.

#### 2.4.5. Biodegradable Water Absorbing Geocomposite (bioWAG)

The principle of operation and the structure of bioWAG are identical to those of the WAG. The difference between the two consists in the materials used, which in the case of bioWAG are 100% biodegradable in the soil environment. bioWAG in the form of a mat is built of 100% needle-punched wool, i.e., nonwoven geotextile, a wooden skeleton of the materials used for the construction of wooden punnets, and a superabsorbent polymer for water absorption, i.e., Aquasorb 3005 KL (SNF Floerger, Andrézieux, France).

#### 2.4.6. Biochar

The commercially available biochar (Fluid S.A. Poland) used in this study was obtained in an innovative process of autothermal thermolysis of miscanthus biomass at 300 °C in an anaerobic atmosphere. The biochar used in this experiment was also used in studies by other authors [118,119]. The characteristics of the biochar were compiled on the basis of information provided by Ścisłowska et al. [119] (Table S2). The air-dried biochar product was not sieved before application.

#### 2.4.7. Superabsorbent Polymer (SAP)

The superabsorbent polymer used in the experiment was Aquasorb 3005 KL (SNF Floerger, Andrézieux, France), which is a commercially availablea cross-linked copolymer of acrylamide and potassium acrylate. As a treatment, it was mixed directly with soil as well as used in the WAG and bioWAG as an integral part of the treatment technology. The presented physical properties of Aquasorb 3005 KL were reproduced from the manufacturer’s specifications (Table S3). An illustration of the chemical structure of the SAP used in the study is presented in Supplementary Materials (Figure S1). Aquasorb 3005 KL has a grain size distribution as follows: 43% of 1.0–2.0 mm range, 30% of 0.5–1.0 mm, 24.5% of 2.0–5.0 mm and 2.5% of 0.25–0.5 mm range [75].

### 2.5. Water Absorption of Used Materials

Water absorption was verified with the use of the gravimetric method. Weighed one-gram samples were soaked with distilled water (temp. 20 °C) until the level of 100% saturation was attained. Next, the unbound water was removed, the samples were weighed and dried successively for 24 h at 105 °C, and then they were weighed again.

### 2.6. Data Analysis

The experiment was conducted in a randomised design with three replicates of each treatment and three repetitions of the wetting-drying cycle. Therefore, the data are presented as means of three sample replicates and three experiment repetitions. Standard errors were calculated. Data distribution and homoscedasticity have been verified (Figures S2–S19). A one-way ANOVA with α = 0.05 significance level was performed to analyse the differences in soil moisture and soil matric potential among soil samples treated with soil amendments. The differences of means were assessed by Tukey’s HSD and Duncan tests at the same significance level α = 0.05. All statistical analyses were performed with the statistical software package Statistica v. 13.3.721.0 (TIBCO Software Inc.).

Data acquired from the experiment conducted using the pressure plate extractor were used to plot the water characteristics of soil treated with soil amendments. With the use of the SWRC Fit software v. 3.0, soil hydraulic models by van Genuchten [120], Seki [121], Durner [122], Kosugi [123], Fredlund and Anqing Xing [124], Brooks and Corey [125] were fitted to the measured soil water retention curves [121]. SWRC Fit software performs nonlinear fitting of curves using the Levenberg–Marquardt method. Ultimately, one fitted model was presented for each of the soil amendments used, based on the lowest Akaike information criterion value. The most appropriate models chosen for the curve estimations were the van Genuchten, Seki and Durner models. Equations with determination parameters were presented in the table below (Table 1). Additionally, the coefficient of determination R^2^ was presented.

The differential porosity of the samples was determined on the basis of the division proposed by Marshall [126]. That division introduced macropores at the level of pF 2.0, which corresponds to a macropore size threshold of 30 µm (>30 µm macro, 30–0.2 µm meso, <0.2 µm micro). Finally, a descriptive statistical analysis was performed to better view the data collected from the pressure plate extractor experiment.

## 3. Results

**Figure 3:**
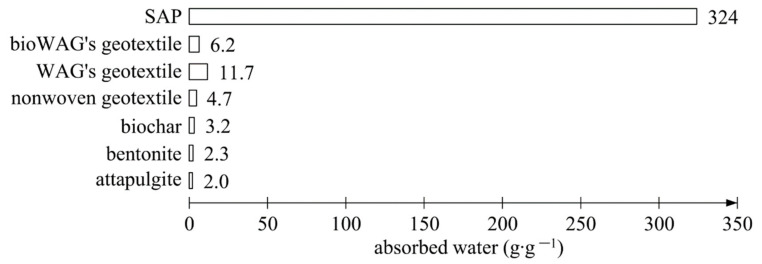
Water absorbency of materials used (*n* = 5). The amount of quantity water is presented in conversion as per one gram of soil amendment.

Before the start of the experiment proper, a preliminary study was conducted to determine the maximum water absorption capacity of the soil amendments chosen for use in the experiment (Figure 3). The superabsorbent polymer was characterised by the highest water absorption capacity among the soil amendments used. In addition, the amounts of water absorbed by the geotextiles, which are an inseparable element of some amendments, such as WAG and bioWAG, were also verified. The synthetic geotextile used in the study absorbed 88.7% more water than the biodegradable textile of 100% wool. With regard to the form of the amendments, only the granular amendments were analysed, taking into account the geotextiles which were components of the WAG and bioWAG solid-state soil amendments.

**Figure 4:**
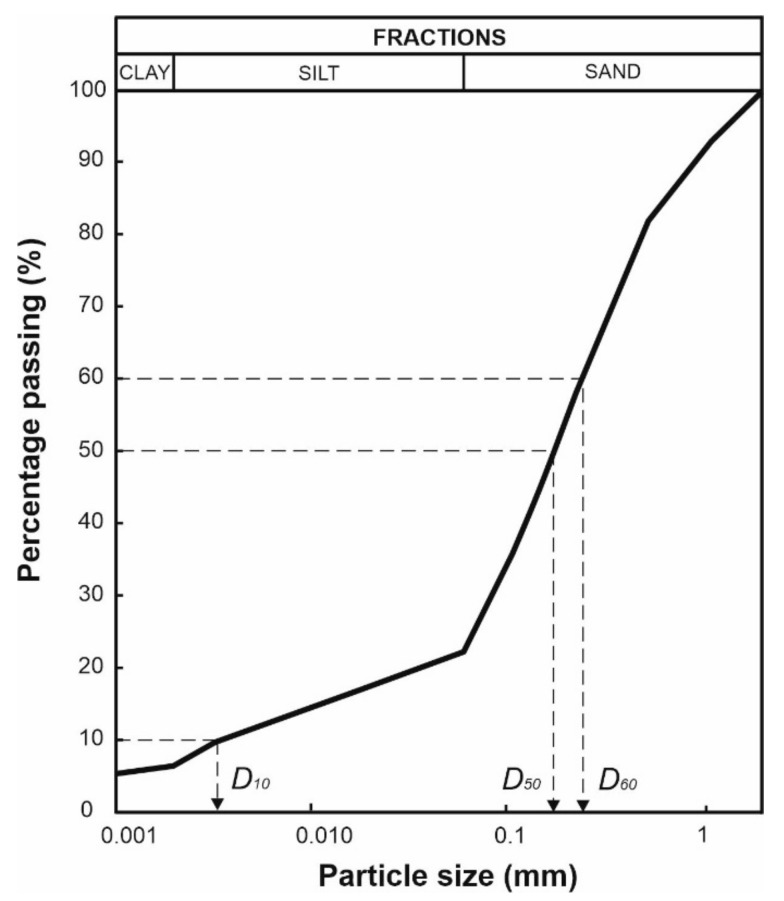
Particle size distribution of soil used in the experiment.

In addition, the particle size distribution and physico-chemical properties of the soil were analysed with the sieve and aerometric methods (Figure 4 and Table 2). Three samples were taken for every soil parameter test, and the results were averaged.

### 3.1. Soil Moisture in Pot Samples

Over almost the entire observation period, the mean values of moisture were higher in the pots with the soil amendments than in the control samples (Table 3 and Figure S20). However, not all the differences were statistically significant. In the samples in which the soil amendments were used, on days 4, 7 and 14, distinct moisture changes were observed in the soil profile, consisting of moisture increases with the depth in the profile. The highest moisture was noted in the bottom parts of the pots and the lowest was in the top layers of soil during the first days of the experiment for most samples. An inverse phenomenon was observed for samples containing SAP. In the control samples, the variation of moisture in the soil profile (top, centre, bottom) was minimal, and from day 7 almost non-existent. The most extensive number of statistically significant differences was noted on the 14th day of measurements. In the final phase of drying of the samples, uniformisation of moisture in the entire soil profile was observed in all of the samples. On the final day of observations, the highest mean values of moisture were noted at the centre depth of the samples containing soil amendments, such as WAG, bioWAG and SAP.

An analysis of the accumulated data revealed that on each day, statistically significant differences were noted between the mean soil moisture in the samples containing WAG and the mean moisture of the control samples at two depths (centre and bottom) (Table 3). In the top layer of the samples containing WAG, the moisture values noted were also higher than in the control samples, but the differences were not statistically significant. In the samples containing WAG, higher mean values of moisture compared to the control samples were observed at every depth in the soil profile. At the same time, WAG was the soil amendment whose application caused statistically significant differences in mean soil moisture relative to the control most frequently throughout the conducted experiment. In the topsoil layer of the treated samples, values statistically significant and higher than in the control were noted only in thr samples containing the nonwoven geotextile.

The application of WAG as a soil amendment resulted in higher soil moisture in the centre and bottom layers, by 5.4 p.p. and 6.4 p.p. on day 4 and by 4.5 p.p. and 8.8 p.p. on day 7, respectively, relative to the control samples. During the study period, better efficiency was noted only in the case of samples containing the nonwoven geotextile, but only in the topsoil layer, which on days 4 and 7 accumulated 6.6 p.p. and 5.4 p.p. more soil moisture, respectively, relative to the control samples. On day 14 of the experiment, the most significant differences in mean moisture between the treated samples and the control sample were noted in the top layer of the samples with bentonite (1.8 p.p.), in the centre layer of the samples with WAG (3.7 p.p), and in the bottom layer of the samples with biochar (4.6 p.p.), bioWAG (4.6 p.p.) and WAG (4.4 p.p.). On day 22, the most significant differences in mean soil moisture between the treated samples and the control sample were noted in the top, centre and bottom layers of the geotextile (1.7 p.p.), WAG (4.8 p.p.) and attapulgite (3.2 p.p.) samples, respectively, again in favour of samples containing the soil amendments.

**Figure 5:**
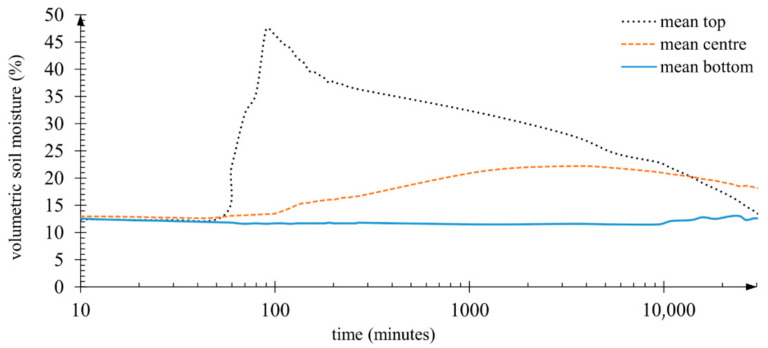
Soil moisture recorded in the course of the wetting–drying process in pot samples containing the mixed soil amendment SAP in the first drying cycle; visible constant moisture level in the bottom layer of the sample (*n* = 3).

In samples containing SAP in the form of a mixture with soil, the moisture in the bottom part of the pot was the lowest among all analysed cases. Moreover, the moisture in the bottom layer of the pots containing SAP did not change over the entire observation period. After the wetting of the samples, water migration to the deepest soil layer was not registered. In the soil’s centre layer, an increase of moisture was recorded only after the maximum moisture level was attained in the top layer. A moisture increase in the centre layer was correlated with a decrease of moisture in the top layer. Interestingly, in minute 200 of observation, the rate of water migration from the top part of the profile to the centre layer was nearly monotonous, at 0.38% of soil moisture per hour. This continued for the first 24 h of measurements. As the effect of this soil amendment on soil moisture was the most atypical and unexpected, it is worth presenting the cycle of moisture changes in samples with the addition of SAP (Figure 5). The graph also shows the moment when, in minute 45 of observation, water was added. As mentioned earlier, the samples were watered with 3 litres of water for 35 min. In the presented graph, soil moisture attained its maximum 45 min after the start of the wetting cycle. The recording interval of soil moisture data was 10 min.

### 3.2. Soil Matric Potential in Pot Samples

Due to the size of the tensiometers, soil matric potential was measured within the entire volume of the top layer of the pots. The results obtained from such measurements were accepted as the value of soil matric potential for the whole pot. The method of probe positioning is illustrated and described in Section 2—Materials and Methods. In an overview of the results obtained, one should note that on days 4 and 7, the differences between the mean values of soil matric potential were minor, except for the samples with the addition of SAP, which differed statistically significantly from the control samples (Table 4). In further observation periods, almost all mean values of pressure differed statistically significantly from the means of the control samples.

On observation days 4 and 7, the highest mean soil matric potential values were recorded in samples containing SAP, at −23.7 kPa and −30.6 kPa, the differences relative to the control being −14 kPa and −18.8 kPa, respectively. On day 14, it was noted that the drastic advantage had evened out, and this time the highest soil matric potential was noted in the samples containing biochar (−68.5 kPa) and WAG (−64.9 kPa), with differences relative to the control being −16.2 kPa and −12.6 kPa, respectively. On day 22, the values of soil matric potential in the samples were nearly uniform, and the highest mean soli matric potential was noted in the samples with an addition of biochar (−76.8 kPa).

Considering high soil matric potential as a stressful soil condition, low soil matric potential as the opposite would be a desirable outcome. Therefore, low but negative soil matric potential was noted only in the samples with nonwoven geotextile treatment. On observation days 4, 7 and 14, the values noted in the samples with the geotextile were −9 kPa, −9.6 kPa and −40.2 kPa. On the final day of observation, the highest soil matric potential registered in a sample with SAP was −63 kPa.

### 3.3. Soil Water Characteristic

**Figure 6:**
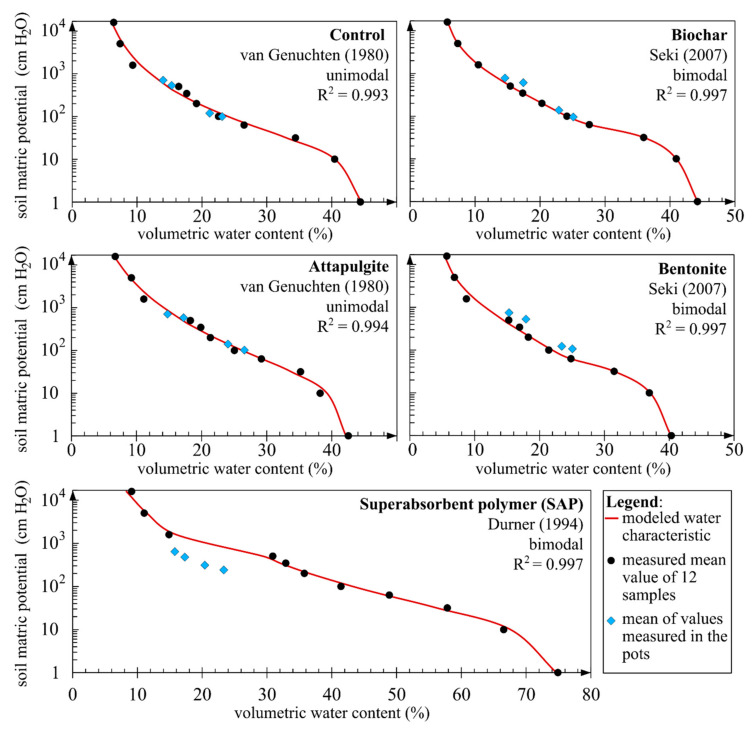
Water retention curves (drying curves) of soil samples (loamy sand) treated with soil amendments. Mean values of soil moisture for the samples from the pot experiment are imposed on the graphs.

To confirm the credibility of the results obtained in the pot experiment, an additional experiment was conducted using a pressure plate extractor. The water retention curve (water characteristic curve) of the samples was obtained for a broader range than was possible to achieve within the scope of the pot experiment. Models of the water retention curve were fitted to the results obtained, achieving very high goodness of fit coefficients (R^2^). In addition, mean values of sample moisture from the pot experiment were imposed on the graphs (Figure 6) as blue squares. It was demonstrated that the results from the pressure plate apparatus were in conformance with those from the pot experiment, with the exception of samples with the additions of bentonite or SAP.

**Figure 7:**
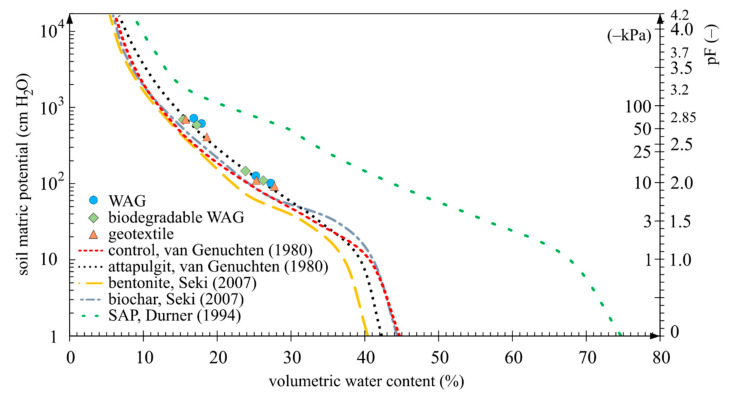
Summary chart of water retention characteristics of soil containing the soil amendments (drying curves) in loamy sand. Mean moisture values of pot samples amended with solid-state amendments such as WAG, bioWAG and nonwoven geotextile are added to the chart. The curves are plotted on the basis of data from the pressure plate apparatus and hydraulic models (*n* = 120).

The analysis of samples with solid-state soil amendments, such as the geotextile, WAG and bioWAG in the pressure plate extractor was not a straightforward task. Those amendments were installed in the soil at a depth of 10 cm. Therefore, instead of analysing those three amendments in the pressure plate extractor, the results representing the behaviour of those soil amendments registered in the pot experiment were used in the summary chart (Figure 7). The obtained mean values of soil matric potential and soil moisture values measured for those amendments tended to concentrate closer to the curve describing the samples containing an addition of attapulgite. Samples that distinctly differed from the others contained an addition of SAP in the form of a mixture with the soil. The difference in pF 2.0 between the sample with the amendment with the highest moisture and the sample with SAP was 17 p.p of soil moisture.

**Figure 8:**
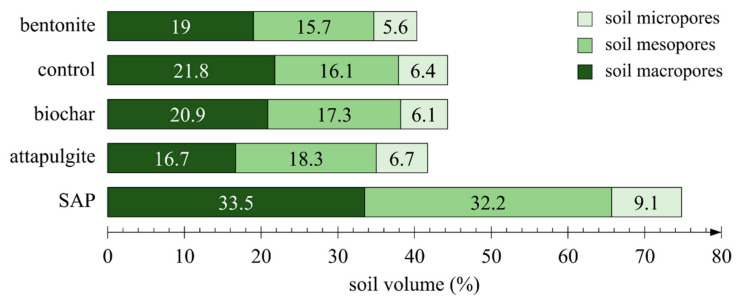
Porosities of soil samples tested in pressure plate extractor. Four samples were taken from the top of every pot to ensure that the samples contained equal amounts of the soil amendments used for soil treatment (*n* = 120).

One aspect that needs to be addressed is the porosity of the samples and the division into micropores, mesopores and macropores in the analysed case, primarily due to the superabsorbent polymer. The method used in the pressure plate extractor experiment requires that samples be 100% saturated with water before they can be placed in the apparatus. Due to the strong swelling effect of the superabsorbent polymer, a part of the soil was forced out of each measuring cylinder containing that amendment before the pressure plate experiment was started. The effect of the addition of the superabsorbent polymer on sample porosity was considerable in comparison to the other soil amendments (Figure 8). The porosity of the remaining samples with the granular amendments was diversified only to a small extent. Excluding SAP, the standard deviations in the groups of micropores, mesopores and macropores were as follows: 0.41%, 1.02% and 1.96%, respectively.

Descriptive statistics of the data accumulated as a result of the pressure extractor plate experiment are provided in Table 5. The standard error obtained for the moisture of samples measured at various pressures can be accepted as satisfactory. With an increase of pressure in the apparatus, the variation of the results obtained usually decreased.

Adopting a threshold value of pF 2.0, at which gravitational water has drained, the amendments such as SAP, biochar and attapulgite retained, on average, more water in the soil than the control samples, by 18.8, 0.9, and 2.4 percentage points, respectively. Conversely, samples containing bentonite had a lower water content than the control samples, on average, by 1.4 percentage points. In the course of the process of drying at a higher pressure value, i.e., pF 2.7, the SAP, biochar and attapulgite retained, on average, more water than the control samples, by 14.5, 0.2 and 1.9 percentage points, respectively. In the samples with an addition of bentonite, at pF 2.7, a lower mean moisture relative to the control sample by 1.1 percentage point was noted.

## 4. Discussion

### 4.1. Superabsorbent Polymer (SAP)

In pot samples with the SAP—Aquasorb 3005 KL treatment, the mean moisture in the 10 cm thick top layer (Top) was higher by 2.3 p.p. than in the control samples until day 4. Until the end of the conducted observations, soil moisture values never attained a level lower than noted in the control sample. Still, in principle, higher values of moisture were expected in the top layer of the soil throughout the observation period. Narjary et al. [81] report that, during the initial 4–7 days, moisture depletion in an Alluvial soil (66% sand, 9% silt, 25% clay) mixed with 0.7% admixture of SAP can be 1.9% of soil moisture per day (depending on the initial water dose and on the rate of evaporation), which is basically in line with our results. On day 4, in the top layer of the samples with SAP, an average soil moisture of the order of 25% was noted, which, according to the fact cited above, should have decreased after 3 days to 19.3%. In fact, the samples containing SAP attained an average moisture of 21.1 p.p. On observation days 7–14, the depletion value decreased to less than 0.93% of soil moisture per day. On day 14 of drying, we should have obtain a value of 14.6%, while it was 16.7% in reality. These differences may have be related to soil bulk density, SAP-applied soil texture, and ambient temperature. Narjary et al. also noted that the most significant depletion of moisture between treated samples and the control took place 4–7 days after watering, at the level of 1 p.p. per day. Galeş et al. [82] conducted a 2-year summer measurement campaign using a similar superabsorbent polymer, but at a considerably smaller amount, 30 kg∙ha^−1^ (Aquasorb 3005A). In a loamy clay texture type soil, they noted a mean difference between the treated sample and the control amounting to barely 1 p.p of soil moisture in the two years of observations, and at a depth of 5–10 cm. It should be mentioned that the field in question for that study was under maize cultivation, with no irrigation, and the soil moisture was measured using the gravimetric method.

Considering soil matric potential, statistically significant differences relative to the control samples were observed on observation days 4, 7, 14 and 22. The values noted on days 4, 7 and 14 were −23.7 kPa, −30.6 kPa and −47 kPa, respectively, and they were the highest values of soil matric potential on those days in the group of the analysed soil amendments. For comparison, the values of soil matric potential in the control samples on the same days were −9.8 kPa, −11.8 kPa and −52.3 kPa, respectively. The obtained soil matric potential values of the samples treated with SAP did not correlate with soil moisture values in those samples, especially if we consider the values of soil matric potential and soil moisture obtained in the control samples. The reason for this could have been the use of a superabsorbent polymer with high hydrophilicity, which generated a high difference of potentials between the porous filter of the tensiometer measuring the matric potential and the particles of the superabsorbent polymer in the conditions of partial saturation of the soil with water. Such a possibility was noted in the study by Saha et al. [127], who suggested that the presence of SAP and its behaviour in soil pores can interfere with the readings of the suction tensiometers.

Surprisingly, in the lowermost layer in which moisture was monitored, no changes were noted in soil moisture content (Figure 5). Over the entire observation period, moisture in the bottom layer remained at the initial level of approximately 12–13%. This was probably the effect of applying a dose of water that the superabsorbent polymer in the top layer of soil could absorb entirely. According to the preliminary experiment, 1 g of the superabsorbent polymer absorbed 324 g of water (Figure 3). Therefore, the theoretical water absorption capacity of the SAP was 14 256 g of water, not including the retention capacity of the top layer of the soil. This is supported by the fact that the samples containing SAP swelled. The surface of the samples rose by about 2 cm, which was the effect of the increased volume of the SAP particles. One can, therefore, wonder at the somewhat high values of soil moisture in the top and centre soil layers measured with the TDR probes. Once again, the explanation should be sought in the high hydrophilicity of the superabsorbent polymer used. Nevertheless, TDR techniques were applied for such measurements in studies estimating the effectiveness of superabsorbent polymers in soil [128,129,130,131]. The authors of these studies did not take note of that problem. A similar relationship concerning the absence of effluent in samples treated with superabsorbent polymer was presented in studies by Hüttermann et al. [69] and Fitch et al. [132]. In addition, Hüttermann et al. [69] observed that samples containing the highest concentrations of superabsorbent polymer lost the greatest amounts of water through evaporation. It seems logical that the more water is accumulated, the more water can evaporate. Hence, the problem may be the dynamics of the process of evaporation due to water being retained at a shallower depth, which implies certain limitations of a practical nature in the use of superabsorbent polymers directly mixed with soil.

In the present experiment, after applying SAP, the bulk density of the samples decreased from 1.70 g∙cm^−3^ (control) to 1.14 g∙cm^−3^ (Table 5). In a study by Baran et al. [133], loamy sand amended with a 0.6% dose of superabsorbent polymer changed its bulk density from 1.29 g∙cm^−3^ to 1.15 g∙cm^−3^. Studies of superabsorbent polymers and their effects on soil usually conclude that the effects are environmentally beneficial, such as reduced bulk density and increased porosity [79,134]. There is usually no mention of the potential effects of the continuous presence of superabsorbent polymers in soil with increased moisture, e.g., during the rainy season or technological irrigation. Swollen SAP reduces soil hydraulic conductivity for periods from several to over a dozen days [135] (depending on the rate of evapotranspiration), which may have an impact on the amount of air in the soil. Therefore, the discussion should instead focus on the wind erosion of topsoil formed in this manner. The swelling behaviour of the superabsorbent polymer Aquasorb 3005 KL was measured experimentally by Lejcuś et al. [75]. Under a load equivalent to that of a soil layer with a thickness of 10 cm and bulk density of 1.30 g∙cm^−3^, SAP can absorb a maximum of 63.4 g∙g^−1^ (amount of water absorbed per one gram of SAP) from watering in 4 h. The same SAP with zero loadings can absorb 338.5 g∙g^−1^ in as little as 63 min. It needs to be mentioned that these values apply to an initial period of SAP use and to swelling in distilled water in the conditions of full saturation under simulated load (not mixed with soil), and therefore actual values of absorbed water can be different, especially with the ageing of SAP in soil. Misiewicz et al. [76] reported that a superabsorbent polymer at a concentration of 0.5% or 1%, mixed with sandy loam (sand 77%, clay 9%, silt 14%) with a bulk density of 1.50 g∙cm^−3^, which was very close to loamy sand by texture, under the load of a soil layer with a thickness of 10 cm, absorbed 85 g∙g^−1^ of water. The described effect of the use of SAP leads to the conclusion that SAP is capable of retaining large amounts of water in the soil that could not be retained naturally, and in the context of the conducted experiment, that the entire amount of supplied water (3 L) was retained in the soil.

**Figure 9:**
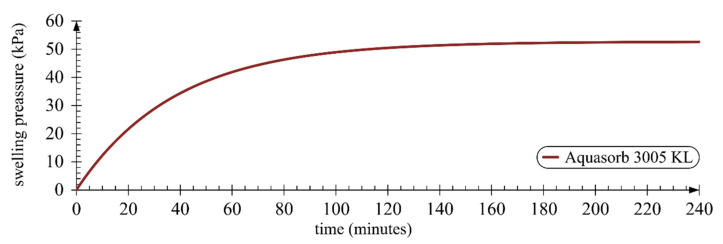
Theoretical swelling pressure for loamy sand soil (sand 83%, silt 10%, clay 7%) in a topsoil layer characterised by soil bulk density of 2.0 g∙cm^−3^ and the superabsorbent polymer Aquasorb 3005 KL in the amount of 0.7% in the first cycle of swelling. The curve was drawn according to the model by Misiewicz et al. [77].

Misiewicz et al. [77] proposed a model that may be used to determine the theoretical swelling pressure generated in loamy sand soil and coarse sand. It is the first version of the model and has a fixed soil type and bulk density, although it allows modification of the concentration of superabsorbent polymer or the choice of a superabsorbent polymer with suitable granulation. Therefore, the proposed model was used to conduct a simulation that determined the theoretical increase of the value of swelling pressure (Figure 9). The theoretical swelling pressure of 50 kPa was attained in minute 112 of the swelling process. Nevertheless, it is necessary to point out certain limitations, such as a change of soil porosity or water absorption capacity over time. Therefore, the presented value should be taken as an approximation that gives an idea of the forces exerted by SAP on the surrounding soil structure and provides an explanation of the changes taking place in the soil structure and moisture. It may be that the value of 50 kPa actually allows the retention of 85 g of water per 1 g of SAP (with zero loading—324 g of water). With a deeper application, that pressure would be insufficient, as would be be reflected in a smaller amount of retained water.

Analysing the results of the additional experiment with the use of the pressure plate extractor in the context of the use of SAP, it was noted that the results obtained for soil moisture and soil matric potential differed from those registered in the pot experiment (Figure 6). Most probably, the cause of the difference was, as mentioned earlier, the amount of water supplied to the samples at the start of the experiment. Each pot sample was watered with three litres of water, which was demonstrated to be a small amount in relation to the maximum absorption capacity of SAP. During the experiment in the pressure plate extractor and sandbox, the swelling superabsorbent polymer pushed out a part of the soil from the measurement cylinders. The experiment was conducted without any sample loading. This phenomenon results from the fact that, in conformance with the method the samples underwent 100% saturation, which ultimately impacted the porosity of the sample and the water characteristics of soil containing such an amendment. The addition of SAP caused increases of porosity in the groups of micropores, mesopores and macropores by 54%, 100% and 42%, respectively, relative to the control. Ultimately, the volume of pores in the sample amounted to 90% (Figure 8). The demonstrated increase in porosity was the highest among the analysed samples. Correspondingly, the samples with the superabsorbent polymer analysed in the pressure plate extractor accumulated notably more significant quantities of water. As an example, at pF 2.4, the difference observed was as much as 11 p.p. relative to data from a soil water characteristic curve plotted on the basis of the pot experiment. Dorraji et al. [73] determined the soil water retention curve for the superabsorbent polymer Superab A200 in a loamy sand soil for an SAP concentration of 0.6% and a suction of −50 kPa and obtained a difference between the amendment and control at the level of 14 p.p. in favour of the sample containing the amendment. In our experiment (pressure plate apparatus), a nearly identical difference was obtained, of the order of 13 p.p., albeit under the altered conditions that a different SAP was used and the soil conductivity was different. Our result was 123 µS∙cm^−1^, and that obtained by Dorraji et al. was 1500 µS∙cm^−1^. In a study conducted by Narjary et al. [81] by the use of pressure plate extractor in an Alluvial soil (66% sand, 9% silt, 25% clay) mixed with a cellulose-based grafted and cross-linked anionic polyacrylate superabsorbent polymer, a 21 p.p. difference between the treated and control samples was found at −50 kPa. For a pressure value of −10 kPa, the difference was 24.4 p.p of soil moisture, while in our experiment, the difference at −10 kPa was 19.3 p.p. of soil moisture. In a study by Abedi-Koupai et al. [136], for loamy sand (sand 77%, silt 10%, clay 13%) with a bulk density of 1.78 g∙cm^−3^, at the pressure of −50 kPa, the moisture of the control sample was at the level of 7.4% while that of a sample with SAP applied at the concentration of 0.8% was at the level of 24.4%. The results obtained in our experiment indicate conformance with the results from the cited studies.

### 4.2. Biochar, Attapulgite and Bentonite

Samples treated with biochar, attapulgite and bentonite permitted the retention of additional amounts of water in the soil. The mean moisture levels in those samples were generally higher than in the control samples. The most significant differences between the treated samples and the control samples were noted on observation days 4 and 7. In the centre layer of the soil, relative to the control, on day 4, the differences noted for the samples containing attapulgite, biochar and bentonite were 4 p.p., 3.7 p.p. and 2 p.p., respectively, and on day 7 they were 3.2 p.p., 3.1 p.p. and 2.1 p.p. However, not all of the differences were statistically significant.

In the top layer of the soil, the differences in soil moisture relative to the control were minor. In the samples containing biochar, the soil moisture in the top layer was almost equal to that of the control samples. Initial water holding abilities were investigated by Novak et al. [137], who experimented with a loamy sand soil (sand 74%, silt 25%, clay 1%) with a bulk density of 1.30 g∙cm^−3^ to 1.40 g∙cm^−3^. Within the scope of the experiment, they added various biochars to the soil, at a concentration of 2%. After flooding the samples with water and waiting for 30 h, differences in the amount of retained water (soil moisture) between the control and samples containing peanut hull (surface porosity 1.22 m^2^∙g^−1^) or poultry litter (surface porosity 9.0 m^2^∙g^−1^) were 2.8 p.p. more and 0.3 p.p less, respectively, in the treated samples compared to the control. In a study conducted by Novak et al. [138] noted that the differences in the water holding capacity between the control and treated samples after 6 days of evaporation were 2.7 p.p., 0.9 p.p. and 4.6 p.p., respectively, for peanut hull (surface porosity 1.22 m^2^∙g^−1^), poultry litter (surface porosity 9.0 m^2^∙g^−1^) and hardwood (surface porosity 1.28 m^2^∙g^−1^, made from wood wastes). They also pointed out that, with an increase in the sample’s bulk density, the water holding capacity decreased. Therefore, despite the larger dose applied, i.e., 2%, and the lower bulk density in samples with various soil amendments, the values of soil moisture in the experiment by Novak et al. were similar to those presented in this study. Novak et al. [138] also determined the water characteristics of biochar treated soil samples. For −50 kPa and −10 kPa, the differences between the control samples and samples treated with hardwood biochar were 0.6 p.p. and 1.3 p.p., respectively. In the case of the biochar produced from peanut hull and poultry litter, the difference was 1.7 p.p. in favour of the treated sample. In our experiment, the differences between the control and biochar treated sample for −10 kPa and −50 kPa were 0 p.p. and 0.7 p.p. of volumetric soil moisture, respectively. Głąb et al. [66] obtained similar results in a study where biochar from *Triticum aestivum L.* and *Miscanthus × giganteus* was applied to loamy sand soil (81% sand, 14% silt, and 5% clay) with a bulk density of 1.60 to 1.70 g∙cm^3^ at the concentration of 0.5%. That study included the use of biochar with various particle size fractions, i.e., 0–500 µm, 500–1000 µm and 1000–2000 µm. In samples containing biochar produced from *Triticum aestivum L.*, at concentrations of 0.5% and 1% and with a granularity of 0–500 µm and 500–1000 µm, at pressures lower than −10 kPa, the values of water retention in the soil were nearly identical to those in the control samples. In samples with biochar concentrations of 0.5% and 1%, and particle size fraction 1000–2000 µm, moisture values higher than the control samples by 1.4 p.p. were noted. Samples treated with biochar from *Miscanthus × giganteus* were characterised by the highest water retention in the soil. For the fraction of 1000–2000 µm, with biochar concentrations of 0.5% and 1%, the differences between the treated samples and the control were 1.2 p.p. and 1.7 p.p., respectively, in favour of the samples with the amendment. The biochar used in our experiment had a particle size fraction of 2000–5000 µm, which may account for the divergence in results. Quin et al. [139] analysed the effect of biochar produced from oil mallee trees (*E. polybractea*), with a specific surface area 269.1 m^2^∙g^−1^, in Arenosol, a sandy soil with 1.3% clay content and a bulk density of 1.6 g∙cm^−3^. For a 1% concetration of oil mallee tree biochar, the differences between the treated samples and the control at pressures of −10 kPa and −50 kPa were 3 p.p. and 0.9 p.p., respectively, in favour of the treated samples, which again is in conformance with the results obtained in the present study.

According to the conclusion from a study by Jahan et al. [65], biochar should be selected before application by considering the properties of the soil to which it is to be applied. The effects achieved following the application of biochar also depend on the kind of biochar used and on the temperature at which it was produced. Jahan et al. claim that the addition of straw biochar (10 and 20 t∙ha^−1^) and rice straw biochar (10 t∙ha^−1^) to sandy loam had a poor influence on water retention and plant growth, and that this type of biochar should not be used for coarse-textured soils. The biochars used were characterised by a surface porosity of 4.7–22.2 m^2^∙g^−1^, where the surface porosity of the soil in control samples was 15.1 m^2^∙g^−1^. Similar limitations are imposed on the application of biochar in soil by the authors of a review article strongly pointing out that future water retention efficiency of biochar used relies on feedstock type and temperature analysis [28]. Moreover, similar conclusions were formulated by Jeffery et al. [57]. However, one should not be too strict in assessments concerning the formation of aggregates during the time, which, after all, might cause an increase in soil moisture [40,140]. As for most other issues, in this case, opposite opinions may be found, e.g., in the study by Aller et al. [50], who demonstrated that old biochars perform less efficiently than fresh ones. Nevertheless, it should be noted that the period of drying lasted for only 30 days, and the biochar was aged artificially in laboratory conditions. This demonstrates that the application of biochar to improve soil functions is not as straightforward as it might appear and requires expert knowledge.

Another granular amendment that was applied in the soil was bentonite. The effects of bentonite application in soil were similar to those of biochar, despite the apparent differences in the origin and operation of those soil amendments. According to the pressure plate apparatus experiment, at pF 2.0, the samples containing bentonite retained less water by 2 p.p. than the samples containing biochar. Initially, that difference was as much as 4 p.p., but as the pressure increased, it settled down. On day 14 of drying, the addition of bentonite allowed the maintainance of more moisture in the top and centre soil layers by 1.8 p.p. and 3.2 p.p., respectively, relative to the control sample. According to Table 5, the soil bulk density changed from 1.7 g∙cm^−3^ to 1.5 g∙cm^−3^, suggesting the scale of the potential swelling of this soil amendment. The porosity of the samples presented in the graph in Figure 8 indicates a decrease in pore volume relative to the control sample. That decrease indicates a reduction in the volume of pores in the individual size fractions, i.e., macropores, mesopores and micropores, relative to the control, by 2.8 p.p., 0.4 p.p. and 0.8 p.p., respectively. In this context, it is interesting to note the phenomenon presented in the graph in Figure 8, which shows that samples containing bentonite in the pot experiment retained more water than the samples analysed in the pressure plate apparatus.

In our study, the difference in soil moisture between samples treated with biochar and bentonite was slight. In the centre layer of the samples with bentonite, on day 7, higher moisture was observed by approx. 1.3 p.p. relative to the samples treated with biochar. In the top layer of the samples treated with bentonite, the moisture was higher by an average of 1.8 p.p. relative to the control. In a study conducted by Alghamdi et al. [32], in sandy soil (sand 95%, silt 2.9%, clay 0.4%) with a bulk density of 1.50 g∙cm^−3^, the difference between samples treated with biochar and with bentonite applied at the concentration of 3% was 1.9 p.p. of soil moisture at a depth of 5 cm in the soil profile, in favour of bentonite. In comparison to the control, the samples treated with bentonite retained more water, by 4.4 p.p., than samples with biochar, where the difference was 2.5 p.p. It should be noted that the values obtained at the concentration of 3% were very close to our results, although in our experiment the amendment was added at the rate of only 0.7%. An interesting study was conducted by Mohawesh and Durner [1], in which the impact of biochar, bentonite and hydrogel amendments on sandy soil (sand 96%, silt 4%, clay 1%) with a bulk density of 1.60 g∙cm^−3^ were analysed. According to the results obtained by those authors, the most effective amendment was hydrogel. The difference between bentonite treatment at the concentration of 0.5% and the control was 3.6 p.p., while in the case of hydrogel it was 18.7 p.p., and in the case of biochar (wood chips) it was as low as 1.2 p.p. at pF 2.0 (−10 kPa). The effect of the bentonite amendment on soil hydraulic parameters was also investigated by Mi et al. [33], in which bentonite’s influence on sandy loam soil (sand 72.8%, silt 13.4%, clay 13.8%) with bulk density 1.40 g∙cm^−3^ was monitored for five years. In the consecutive years, soil moisture in the samples treated with bentonite at the rate of 6 t∙ha^−1^ was higher than in the control samples by 0.25 p.p., 0.3 p.p., 0.3 p.p., 0.2 p.p and 0.16 p.p., respectively. The effect of bentonite treatment at the rate of 12 t∙ha^−1^ on soil moisture was also slight. In the consecutive years, the samples with bentonite applied at the rate of 12 t∙ha^−1^ were characterised by 0.8 p.p., 0.7 p.p., 0.5 p.p., 0.4 p.p. and 0.3 p.p. higher moisture, respectively, relative to the control. The samples were collected from a depth of approx. 5 cm and the soil moisture was measured with the gravimetric method. In our study, the effects obtained at the concentration of 8.8 t∙ha^−1^ were more favourable, although the time between water distribution and measurement was shorter.

The application of attapulgite permitted the retention of additional amounts of water in the soil. On the fourth day of observations, the difference in soil moisture relative to the control was 4 p.p. and 3 p.p., in the centre and topsoil layers, respectively. On the seventh day, the mean moisture values obtained should still be considered as satisfactory, as they were 3 p.p. and 2.5 p.p. higher relative to the control in the centre and topsoil layers, respectively. The obtained values of soil matric potential in the treated samples were nearly identical to those in the control samples. According to the results obtained in the pressure plate apparatus experiment, the difference between the control and the sample with attapulgite at −10 kPa was 2.3 p.p. of soil moisture. At the pressure value of −50 kPa, that difference was 2.4 p.p. Among the analysed soil amendments of mineral origin, attapulgite proved to be the best in maintaining moisture in the soil. In addition, in samples treated with attapulgite, there was a reduction of the number of pores, which most probably resulted in an increase of soil bulk density from 1.70 g∙cm^−3^ to 1.75 g∙cm^−3^, due to the application of attapulgite in the form of pulverised powder. Although large amounts of attapulgite have been sold on the market and numerous studies have been published on its application in agriculture, it is still challenging to find a study in which pure attapulgite has been used. According to current trends, attapulgite is applied jointly with other treatments, e.g., fertilisers, to improve the physicochemical parameters of soil.

### 4.3. Nonwoven Geotextile, WAG, bioWAG

In samples containing WAG and bioWAG, higher moisture levels were noted relative to the control samples over almost the entire observation period. In the top layer of the samples, the differences were slight and thus statistically insignificant. Soil samples treated with WAG accumulated the largest amounts of water in the centre layer of the pot on every analysed day. The operation of bioWAG was more balanced. On average, the addition of WAG caused the retention of larger amounts of water compared to the other amendments, although the differences between the mean values in the samples with the remaining amendments were usually statistically significant. The effect of nonwoven geotextiles on the distribution of moisture in the soil profile can be described differently. In in the top layer of the soil of the samples with the geotextile significant differences were noted relative to the control samples against the background of all of the analysed soil amendments (Table 6). This phenomenon can be attributed both to the nonwoven geotextile wicking abilities of WAG’s geotextile and the capillary break phenomenon.

As for samples containing only nonwoven geotextile, the presence of the geotextile led to the appearance of the capillary break phenomenon in the soil profile. As a result, a considerable amount of water was retained in the layer above the geotextile, as the complete and undisturbed transfer of water from the layer above the nonwoven geotextile layer to the soil layer beneath it requires the matric potential on the soil-geotextile interface to be higher than −1 kPa [104]. According to Bouazza et al. [141], a negative suction force between 0.8 and 1.2 kPa is sufficient to induce a rapid decrease of hydraulic conductivity in unsaturated conditions. The reason for this lies in the air entry value, which for nonwoven geotextile varies in the range of 0.4–0.9 kPa [141] or 0.4–1.2 kPa of negative pressure [103]. Perhaps also, for this reason, the geotextiles used in the WAG and individually as the nonwoven geotextile should have been placed in the soil in a saturated state. However, as the remaining soil amendments were used in the experiment in a dry state, it was decided to apply the geocomposite in the dry form, which might have impacted the performance of the WAG and the bioWAG. On the other hand, in the later exploitation period, wetting of dry geomaterial often leads to air entrapment in the larger pores [101].

In the conducted experiment, soil matric potential data were logged at 10-min intervals. The initial value of soil matric potential registered after watering the samples, was −3.2 kPa. Due to this, the state of balance of soil matric potential, allowing water transfer, appeared for a short period of time and ceased after the threshold value of −1 kPa was exceeded. Hence, large amounts of water accumulated in the top layer samples containing nonwoven geotextile. In the samples with WAG, the lowest recorded value was −2.5 kPa. Nevertheless, it must be kept in mind that WAG uses a geotextile with wicking ability. The soil moisture in the top layer of samples containing WAG was lower relative to the centre layer. Despite those wicking abilities, the soil matric potential was relatively stable, although on day 14 its value was about 12.6 kPa higher, relative to the control. This kind of impact of WAG on moisture conservation probably resulted from the difference of the potential generated by the wicking properties of the nonwoven geotextile used. Such an interaction is generally positive, and it constitutes the philosophy of WAG operation, which is water absorption from the environment for later use by plants.

In a study by Śpitalniak et al. [90], it was demonstrated that WAG placed at a depth of 10 cm in a loamy sand soil, after full soil saturation, constituted a barrier for upper water movement even in the situation of the long-term heating of the soil surface (40 °C for 72 h). Because of the application of WAG in the soil profile at a 10 cm depth in loamy sand, the treated samples lost 46% less water than the control samples [90]. In addition, a distinct effect of the wicking abilities of the applied geotextile was observed, which manifested as a difference in moisture between the loamy sand soil at the interface with WAG and the topsoil layer above the geocomposite, of the order of 1 p.p., and in the case of a sandy loam soil by as much as 2 p.p. Nevertheless, despite the wicking abilities of WAG, evaporation from such a geocomposite is possible, though limited. Lejcuś et al. [142] conducted an experiment with the aim to estimate the loss of water from sandy soil and a loamy soil treated with WAG through evaporation. Daily measurements of the mass of the pots demonstrated that after 10 days of evaporation, the control samples lost 59% of water, while the samples containing WAG lost only 10%, in both cases. Therefore, it can be concluded that WAG significantly reduced the evaporation of water from the soil. The studies by Śpitalniak et al. [90] and Lejcuś et al. [142] indicate that the geocomposite can retain considerable amounts of water in the soil profile through water retention inside the WAG, as well as through interaction with the soil environment.

Additions of WAG, bioWAG and the nonwoven geotextile had no impact on the change of soil porosity or soil bulk density. Finally, water retained in WAG was not taken into account because the water budget was not analysed. Water retained in WAG has a significant influence on the total water budget in the soil profile, which is an absolute advantage of WAG [90]. The other soil amendments have limited water retention possibilities within their structures.

### 4.4. Limitations of the Study

The limitation of this study is that the experiment was conducted in laboratory conditions on one type of soil without plant cover. Further research will be conducted in the form of field trials on different soil types, and with plants.

## 5. Conclusions

The findings from this study show that water absorbing geocomposite (WAG) is a soil amendment that allows the retention of considerable amounts of water in the soil profile. Samples containing WAG had higher water holding capacity at −10 kPa than samples with biochar, attapulgite, bentonite, bioWAG and control by 3.6, 2.1, 5.7, 1 and 4.5 percentage points, respectively. During the evaporation period at 30 °C, WAG proved to be a soil amendment which, on average, retained the most water among all the examined soil amendments. In the WAG samples, up to the 7th day of observation, the moisture content in the middle layer of the pot remained 4.5 percentage points higher than in the control samples. The other soil amendments used in the experiment also caused the retention of additional amounts of water in the soil relative to the control samples. However, only in the case of WAG, the differences obtained relative to the control were statistically significant at two measurement depths on each day of observations.Besides the retention of more significant amounts of water in the soil, the application of the soil amendments permitted evaporation delay. Water migrated more slowly from the layers in which the amendment was positioned and also from the layers positioned beneath the amendments. The application of the amendments in the soil modified the structure of the soil and, indirectly, the soil matric potential necessary to initiate water movement.

Among the analysed soil amendments, the highest water absorption capacity was noted in the case of the superabsorbent polymers. In the soil of the pot samples, SAP absorbed more water than the other amendments used in the experiment. Only samples containing superabsorbent polymers and samples with nonwoven geotextiles had water holding capacity at −10 kPa higher than WAG, by 14.3 and 0.1 percentage points, respectively. However, the volume of pores in the samples with SAP amounted to 90% after examination in the pressure plate apparatus. One should keep in mind that the efficiency of SAP can be significantly reduced. Such limitations result primarily from soil load [75,76] and the content of ions in the soil, as those factors directly affect the performance of SAP [73]. Additionally, due to its hydrophilicity, SAP can lead to soil drying in the area where it was applied, as was observed in our experiment. In spite of the absorption of all available water, the measured values of moisture did not differ significantly from the moisture of the control samples. Shallow positioning of SAP entails accelerated evaporation of water from the soil and deeper placement decreased SAP performance.

The solution which permits the optimum utilisation of the power of superabsorbent polymers was the water retaining geocomposite (WAG), whose structure, allowed for the undisturbed operation of the superabsorbent polymers. The geotextile with wicking abilities used in WAG enhances its efficiency by drawing water into its interior and protecting it against premature evaporation or infiltration into the depth of the soil profile. As a soil amendment, WAG can be used in agriculture, horticulture, soil remediation and engineering applications as erosion control as a good alternative to existing soil amendments. WAG technology limits the consequences of water scarcity in the soil through effective management of water resources. In times of progressing climate change, an increased demand for technologies supporting plant vegetation and conserving water resources such as WAG is forecast.

If we adopt water retention efficiency as the primary criterion in the classification of soil amendments, SAP, WAG and bioWAG are currently the best known soil amendments for retaining water in soil. Due to the appearance of the new generation of soil amendments, such as WAG and SAP, it seems reasonable to introduce a classification of soil amendments into active and passive categories. Passive soil amendments are those whose presence in the soil does not cause any spatial changes of soil moisture within the area of application of a given amendment, whereas active soil amendments are those whose presence causes measurable water movement in the soil. What is more, water is released from such soil amendments in response to the demand of plants that are their intended recipients, and water is not released before such a demand appears. Water uptake from such amendments occurs through the plant roots that come into direct contact with the soil amendment applied. Therefore, soil amendments, such as SAP, WAG and bioWAG can be classified as active amendments, while biochar, attapulgite or bentonite can be classified as passive.

## 6. Patents

The presented results were obtained on patent use: “Geocomposite element, particularly for enhancing plant growth”, EP2560472, PL211198, which was commercialized in 2012.

## Figures and Tables

**Table 1:** Equations and specific model parameters.

Model/Soil Amendment	Equation	Parameters	R^2^	AIC
van Genuchten (1980) control sample	$S_{e}={\left[\frac{1}{1+{\left(\alpha h\right)}^{n}}\right]}^{m}\;\;\left(m=1-\frac{1}{n}\right)$	$\theta_{s}=0.44811$ $\theta_{r}=0.027197$ α=0.059739 n=1.3685	0.9933	−92.964
van Genuchten (1980) attapulgite	$S_{e}={\left[\frac{1}{1+{\left(\alpha h\right)}^{n}}\right]}^{m}\;\;\left(m=1-\frac{1}{n}\right)$	$\theta_{s}=0.42359$ $\theta_{r}=0.00010024$ α=0.050769 n=1.2791	0.9936	−95.325
Seki (2007) biochar	$S_{e}=w_{1}Q\left[\frac{ln\left(\frac{h}{h_{m1}}\right)}{\sigma_{1}}\right]+\left(1-w_{1}\right)Q\left[\frac{ln\left(\frac{h}{h_{m2}}\right)}{\sigma_{2}}\right]$	$\theta_{s}=0.44732$ $\theta_{r}=0.048397$ $w_{1}=0.18230$ $h_{m1}=45.248$ $\sigma_{1}=0.32746$ $h_{m2}=171.59$ $\sigma_{2}=2.3686$	0.9967	−94.171
Seki (2007) bentonite	$S_{e}=w_{1}Q\left[\frac{ln\left(\frac{h}{h_{m1}}\right)}{\sigma_{1}}\right]+\left(1-w_{1}\right)Q\left[\frac{ln\left(\frac{h}{h_{m2}}\right)}{\sigma_{2}}\right]$	$\theta_{s}=0.40836$ $\theta_{r}=0.042061$ $w_{1}=0.18251$ $h_{m1}=39.537$ $\sigma_{1}=0.39308$ $h_{m2}=177.98$ $\sigma_{2}=2.5162\;$	0.9974	−99.491
Durner (1994) superabsorbent polymer (SAP)	$S_{e}=w_{1}{\left[\frac{1}{1+{\left(\alpha_{1}h\right)}^{n_{1}}}\right]}^{m1}+\left(1-w_{1}\right){\left[\frac{1}{1+{\left(\alpha_{2}h\right)}^{n_{2}}}\right]}^{m2}$	$\theta_{s}=0.75162$ $\theta_{r}=0.012268$ $w_{1}=0.90759$ $\alpha_{1}=0.071400$ $n_{1}=1.3199$ $\alpha_{2}=0.00099505$ $n_{2}=42.733$	0.99730	−85.345

AIC—Akaike information criterion value.

**Table 2:** Soil characteristics.

Parameter	Unit	Method	Soil Type: Loamy Sand (LS)
Silt	%	sieve and aerometric analysis	15
Clay	%		7
Sand	%		78
pH_KCl_	(–)	PN-ISO 10390:1997(A)	7.4 ± 0.3
Total Nitrogen (N)	% DM	ICP-AES	0.06
Potassium (K)	g∙kg^−1^ DM	ICP-AES	1.85
Phosphorus (P)	g∙kg^−1^ DM	ICP-AES	0.519
Magnesium (Mg)	g∙kg^−1^ DM	ICP-AES	1.84
Calcium (Ca)	g∙kg^−1^ DM	ICP-AES	2.45 ± 0.49
Sodium (Na)	g∙kg^−1^ DM	ICP-AES	0.107 ± 0.021
Cation Exchange Capacity (CEC)	cmol(+)∙kg^−1^	only base cations (Ca^2+^ + Mg^2+^ + K^+^ + Na*^+^*)	32.8
Electric Conductivity (EC)	µS∙cm^−1^	PN-ISO 11265+AC1:1997	123.0
Soil organic carbon (SOC)	%	thermal method acc. to PN-88/B-04481	2.86
Bulk density	g∙cm^−3^	Kopecky’s cylinder method	1.67
Multi-fraction soil	(–)	EN ISO 14688-2:2018	C_u_ = 46.67

DM—dry matter.

**Table 3:** Mean soil moisture values in analysed drying cycles.

Day	Depth	SAP	Bentonite	WAG	Biochar	BioWAG	Geotextile	Attapulgite	Control
(–)	(–)	(%)	(%)	(%)	(%)	(%)	(%)	(%)	(%)
4	top	25.0 (0.4)	24.5 (0.9)	25.3 (0.7)	23 (0.9)	25.5 (1.0)	29.2 (1.3) *d	25.6 (0.6)	22.7 (1.8)
	centre	21.6 (0.7)	25.7 (0.7)	28.9 (1.4) *d	27.2 (0.8) d	26.6 (0.6)	26.0 (0.6)	27.5 (1.3) d	23.5 (1.6)
	bottom	11.8 (0.4) *d	30.3 (0.5) d	32.7 (1.6) *d	30.2 (0.3) d	29.1 (1.6)	29.0 (1.0)	29.3 (0.7)	26.2 (0.9)
7	top	21.1 (0.6)	23.1 (0.8)	23.9 (0.8)	21.0 (1.0)	23.2 (0.9)	26.1 (1.1) *	23.2 (0.6)	20.7 (1.7)
	centre	19.6 (0.8)	23.8 (0.7)	26.2 (0.9) *	24.8 (1.0)	24.2 (0.5)	24.2 (0.6)	24.9 (1.2)	21.7 (1.5)
	bottom	12.2 (0.4) *d	27.1 (0.5) *d	29.5 (1.6) *d	26.5 (0.6) *d	26.1 (1.2) *d	25.6 (0.7) *d	26.0 (0.8) *d	20.7 (1.0)
14	top	16.7 (0.3)	17.4 (0.5)	16.6 (0.2)	16.1 (1.0)	15.8 (0.4)	18.9 (0.6) *	17.5 (0.3)	15.5 (1.2)
	centre	17.8 (0.7) d	18.4 (0.5) *d	18.9 (0.8) *d	18.7 (0.8) *d	18.4 (0.4) *d	17.9 (0.7) d	17.2 (0.7) d	15.2 (0.4)
	bottom	12.8 (0.4) *d	18.6 (0.4) *d	19.4 (0.7) *d	19.6 (0.3) *d	19.6 (0.6) *d	18.6 (0.4) *d	19.1 (0.4) *d	15.0 (0.3)
22	top	13.9 (0.3)	14.5 (0.2)	14.4 (0.1)	13.4 (0.7)	13.9 (0.1)	15.6 (0.3)	14.9 (0.1)	13.9 (1.0)
	centre	17.6 (0.6) *d	16.1 (0.7) d	18.9 (0.8) *d	15.8 (0.5)	16.6 (0.4) d	15.5 (0.6)	14.6 (0.3)	14.2 (0.2)
	bottom	13.1 (0.5)	15.8 (0.2) *d	16.3 (0.2) *d	16.2 (0.2) *d	16.3 (0.4) *d	15.6 (0.1) *d	16.6 (0.2) *d	13.4 (0.3)

*—statistically significant difference between means of the treated and control samples following the Tukey test (α = 0.05). d—statistically significant difference between means of the treated and control samples based on Duncan test (α = 0.05). Numbers in parentheses represent standard error.

**Table 4:** Mean soil matric potential values in analysed drying cycles.

Day	SAP	Bentonite	WAG	Biochar	BioWAG	Geotextile	Attapulgite	Control
(–)	(kPa)	(kPa)	(kPa)	(kPa)	(kPa)	(kPa)	(kPa)	(kPa)
Day 4	23.7 (0.6) *	10.5 (0.6)	10.0 (0.4)	9.4 (0.6)	10.8 (0.7)	9.0 (0.4)	10.0 (0.6)	9.8 (0.9)
Day 7	30.6 (0.8) *	12.2 (0.8)	12.4 (0.9)	13.6 (1.4)	14.4 (1.1)	9.6 (0.5)	13.9 (0.7)	11.8 (1.1)
Day 14	47.0 (1.3) d	52.2 (1.4)	64.9 (1.5) *d	68.5 (1.6) *d	57.6 (1.1) d	40.2 (2.1) *d	57.3 (1.3) d	52.3 (1.8)
Day 22	63.0 (1.4) d	73.6 (2.9) d	70.4 (2.1) *d	76.8 (1.2) *d	67.6 (2.3) d	68.4 (2.1) *d	70.1 (1.6) d	69.0 (2.1)

*—statistically significant difference between means of the treated and control samples following the Tukey test (α = 0.05). d—statistically significant difference between means of the treated and control samples based on Duncan test (α =0.05). Numbers in parentheses represent standard error.

**Table 5:** Descriptive statistics of data acquired as a result of the pressure plate extractor experiment.

	pF	Obvs.	Median Moisture (%)	Mean Moisture (%)	Min. Moisture (%)	Max. Moisture (%)	SE in Moisture	Mean Bulk Density (g∙cm^−3^)	SE in Bulk Density
superabsorbent polymer (SAP)	0.0	12	74.1	74.9	69.7	82.5	1.1	1.14	0.020
	1.0	12	66.6	66.5	61.2	73.8	0.9		
	1.5	12	57.3	57.8	52.6	63.1	1.0		
	1.8	12	48.0	48.9	44.2	53.4	1.0		
	2.0	12	40.9	41.4	37.2	45.6	0.9		
	2.3	12	35.6	35.8	31.6	40.3	0.8		
	2.5	12	33.1	32.9	28.7	37.0	0.8		
	2.7	12	31.2	30.9	27.1	34.6	0.8		
	3.3	12	14.2	14.9	10.6	21.9	0.9		
	3.7	12	11.0	11.0	8.9	13.7	0.4		
	4.2	12	9.3	9.1	5.5	12.2	0.7		
control	0.0	12	44.4	44.4	42.2	46.3	0.4	1.70	0.022
	1.0	12	40.3	40.5	39.2	42.2	0.2		
	1.5	12	34.5	34.4	33.2	35.4	0.2		
	1.8	12	26.7	26.5	25.0	27.3	0.2		
	2.0	12	22.8	22.6	21.2	23.4	0.2		
	2.3	12	19.2	19.2	17.5	20.8	0.3		
	2.5	12	17.6	17.7	15.9	18.8	0.3		
	2.7	12	16.2	16.4	15.2	17.6	0.2		
	3.3	12	9.6	9.4	8.0	10.1	0.2		
	3.7	12	7.5	7.4	5.7	8.3	0.2		
	4.2	12	6.6	6.4	3.7	7.6	0.3		
biochar	0.0	12	44.4	44.4	42.6	46.5	0.3		
	1.0	12	40.8	40.9	39.5	42.6	0.3		
	1.5	12	36.0	35.9	34.8	37.5	0.2		
	1.8	12	27.8	27.7	26.3	28.9	0.2		
	2.0	12	23.5	23.5	22.0	25.2	0.2		
	2.3	12	20.0	20.0	18.3	21.1	0.2	1.65	0.019
	2.5	12	18.4	18.3	17.1	20.1	0.2		
	2.7	12	16.3	16.6	15.6	18.4	0.2		
	3.3	12	8.7	8.8	8.1	9.8	0.1		
	3.7	12	7.3	7.4	6.5	8.2	0.1		
	4.2	12	6.3	6.2	5.0	7.8	0.3		
attapulgite	0.0	12	41.7	42.5	39.5	46.0	0.6		
	1.0	12	38.1	38.2	35.6	41.0	0.5		
	1.5	12	35.5	35.2	33.3	36.4	0.3		
	1.8	12	29.4	29.2	27.1	30.0	0.2		
	2.0	12	25.2	25.0	23.2	26.2	0.2		
	2.3	12	21.7	21.3	19.4	22.5	0.3	1.75	0.021
	2.5	12	19.9	19.9	18.4	21.6	0.3		
	2.7	12	18.5	18.3	16.5	20.1	0.2		
	3.3	12	11.2	11.1	10.2	12.2	0.2		
	3.7	12	9.2	9.2	7.4	11.4	0.3		
	4.2	12	6.8	6.7	5.4	7.7	0.2		
bentonite	0.0	12	39.5	40.4	36.1	46.0	0.8		
	1.0	12	36.2	37.0	34.2	41.5	0.6		
	1.5	12	31.1	31.5	29.8	34.7	0.4		
	1.8	12	24.6	24.9	23.9	27.6	0.3		
	2.0	12	21.2	21.4	20.6	23.5	0.2		
	2.3	12	18.3	18.3	16.9	19.8	0.2		
	2.5	12	16.8	16.9	15.6	18.2	0.2	1.50	0.025
	2.7	12	15.1	15.3	14.0	17.2	0.3		
	3.3	12	8.5	8.7	7.6	10.3	0.3		
	3.7	12	6.6	6.9	6.2	8.5	0.2		
	4.2	12	5.7	5.7	4.7	6.8	0.2		

**Table 6:** Differences in mean soil moisture and mean soil matric potential between treated pot sample and control sample.

			Day 4			Day 7			Day 14			Day 22	
Depth	Unit	Gtex	bioWAG	WAG	Gtex	bioWAG	WAG	Gtex	bioWAG	WAG	Gtex	bioWAG	WAG
Top	p.p.	6.6	2.8	2.7	5.4	2.5	3.3	3.4	0.3	1.1	1.7	0.0	0.6
centre	p.p.	2.4	3.1	5.4	2.4	2.4	4.5	2.8	3.2	3.7	1.4	2.4	4.8
bottom	p.p.	2.8	2.8	6.4	4.9	5.4	8.8	3.7	4.6	4.4	2.2	2.9	2.9
SMP	kPa	0.8	−1.0	−0.2	2.1	−2.6	−0.6	12.1	−5.3	−12.6	0.6	1.5	−1.4

Gtex—nonvoven geotextile, p.p.—percentage points.

## Data Availability

The data that support the findings of this study are available from the corresponding author upon reasonable request.

## Supplementary Materials

The following are available online at https://www.mdpi.com/article/10.3390/ma14216658/s1, Table S1: Chemical composition of attapulgite and granular bentonite determined with the use of XRD device.; Table S2: Biochar properties of a dry sample [117].; Table S3: Characteristics of the superabsorbent polymer based on producer information [118].; Figure S1: Chemical structure of superabsorbent polymer Aquasorb 3005 KL based on producer information [118].; Figure S2: Distribution of soil moisture data recorded on the 4th day.; Figure S3: Distribution of soil moisture data recorded on the 7th day.; Figure S4: Distribution of soil moisture data recorded on the 14th day.; Figure S5: Distribution of soil moisture data recorded on the 22nd day.; Figure S6: The homoscedasticity of residuals grouped by soil amendments on the 4th day.; Figure S7: The h homoscedasticity of residuals grouped by soil amendments on the 7th day.; Figure S8: The homoscedasticity of residuals grouped by soil amendments on the 14th day.; Figure S9: The homoscedasticity of residuals grouped by soil amendments on the 22nd day.; Figure S10: Distribution of soil matric potential (SMP) data recorded on the 4th day.; Figure S11: Distribution of soil matric potential (SMP) data of SAP samples recorded on the 4th day.; Figure S12: Distribution of soil matric potential (SMP) data recorded on the 7th day.; Figure S13: Distribution of soil matric potential (SMP) data of SAP samples recorded on the 4th day.; Figure S14: Distribution of soil matric potential (SMP) data recorded on the 14th day.; Figure S15: Distribution of soil matric potential (SMP) data recorded on the 22nd day.; Figure S16: The homoscedasticity of residuals grouped by soil amendments on the 4th day.; Figure S17: The homoscedasticity of residuals grouped by soil amendments on the 7th day.; Figure S18: The homoscedasticity of residuals grouped by soil amendments on the 14th day.; Figure S19: The homoscedasticity of residuals grouped by soil amendments on the 22th day.; Figure S20: Mean soil moisture of pot samples at three depths of measurement (bottom, centre, top). Observations were taken during 22-day long drying cycles. Mean values were based on values of 3 sample replications and three repetitions of the experiment.
