# Supplementary material for: The Assessment of Water Retention Efficiency of Different Soil Amendments in Comparison to Water Absorbing Geocomposite
Source: Materials (Basel). 2021 Nov 4;14(21):6658. doi: 10.3390/ma14216658 (PMC8587147; doi:10.3390/ma14216658)
Supplement: Supplementary file 1 [file materials-14-06658-s001.zip › materials-1289064-supplementary.pdf]

materials

Supplementary Materials

The Assessment of Water Retention Efficiency of Different Soil Amendments in Comparison to Water Absorbing Geocomposite

Michał Śpitalniak [1,*], Adam Bogacz [2] and Zofia Zięba [3]

Table S1. Chemical composition of attapulgite and granular bentonite determined with the use of XRD device.

Parameter	Unit	Limit of Detection	Attapulgite	Bentonite
FeO	mg·kg^{-1}	40	17000	29000
SiO_2	mg·kg^{-1}	0.9	420000	430000
Al_2O_3	mg·kg^{-1}	0.9	110000	54000
Fe_2O_3	mg·kg^{-1}	0.9	46000	83000
CaO	mg·kg^{-1}	0.9	99000	64000
MgO	mg·kg^{-1}	0.9	110000	22000
Na_2O	mg·kg^{-1}	0.9	660	10000
K_2O	mg·kg^{-1}	0.9	6100	6600
TiO_2	mg·kg^{-1}	0.9	580	-
P_2O_5	mg·kg^{-1}	0.9	19000	2200
Chlorine	mg·kg^{-1}	100	-	U/S
pH	-	-	9.1	9.3
Total moisture	%	0.5	5.3	5.1
Analytical moisture	%	0.5	4.3	2.8

Table S2. Biochar properties of a dry sample [117].

Total Moisture (%)	Ash Content (%)	Calorific Value (kJ·kg^{-1})	Carbon (%)	Total Volatile Fractions (%)	Total Sulphur (%)	Hydrogen (%)	Open Porosity (%)	Specific Surface (m2·g^{-1})	Density (g·cm^{-3})
1.7	16.5	28 026	78.00	16.5	0.00	3.00	27.68	1.64	0.71

Table S3. Characteristics of the superabsorbent polymer based on producer's information [118].

Parameter	Characteristic
Chemical composition	Cross-linked copolymer of acrylamide and potassium acrylate
Appearance	dry: white powder<br>hydrated: transparent gel
Particle size	powders, micro granules, granules
pH	8.10
Dry matter	85%-90%
Apparent density	0.85
Specific weight	1.10 g·cm-3
Maximum water absorption (w/w)	400 in deionised water and 150 in soil
Water retention capacity at pF1	980 ml·l-1
Water retention capacity at pF4.2	95%
Cationic Exchange Capacity (CEC)	4.6 meq·g-1
Effectiveness in soil	up to 5 years
Toxicity in soil	none under normal conditions of use

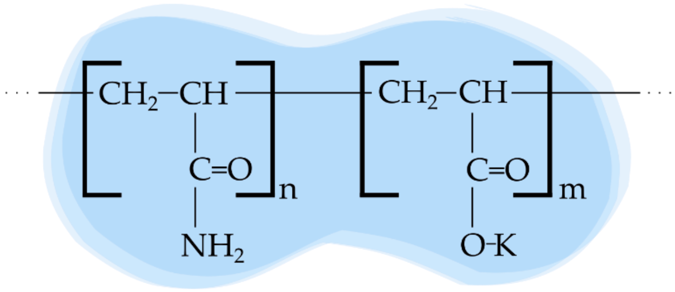

Figure S1. Chemical structure of superabsorbent polymer Aquasorb 3005 KL based on producer information [118].

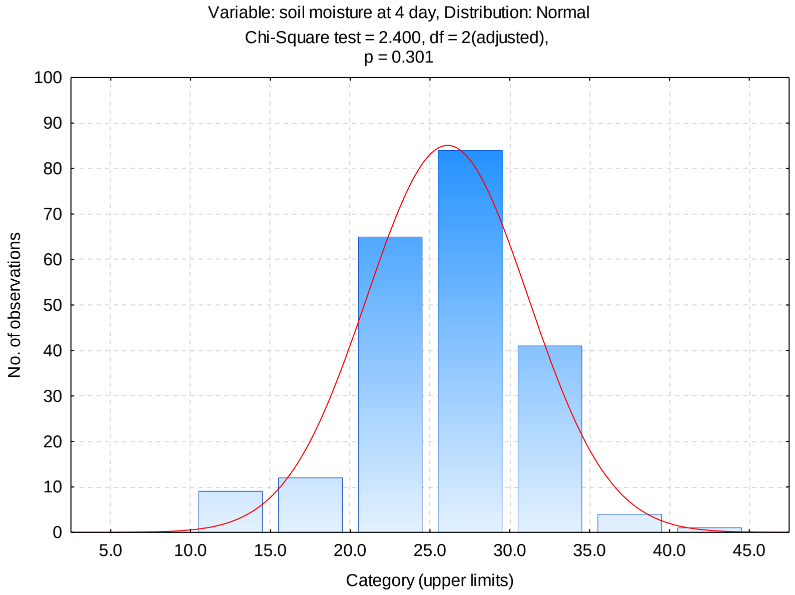

Figure S2. Distribution of soil moisture data recorded on the 4th day. .

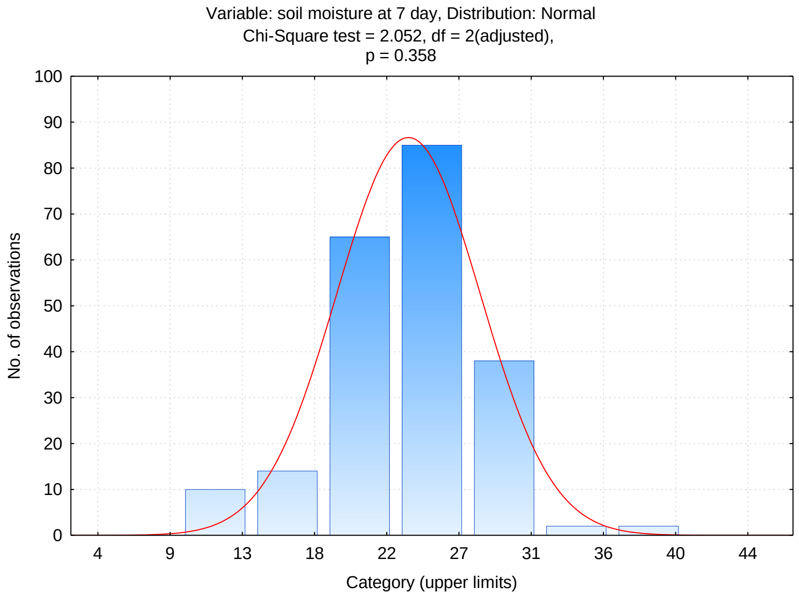

Figure S3. Distribution of soil moisture data recorded on the 7th day. .

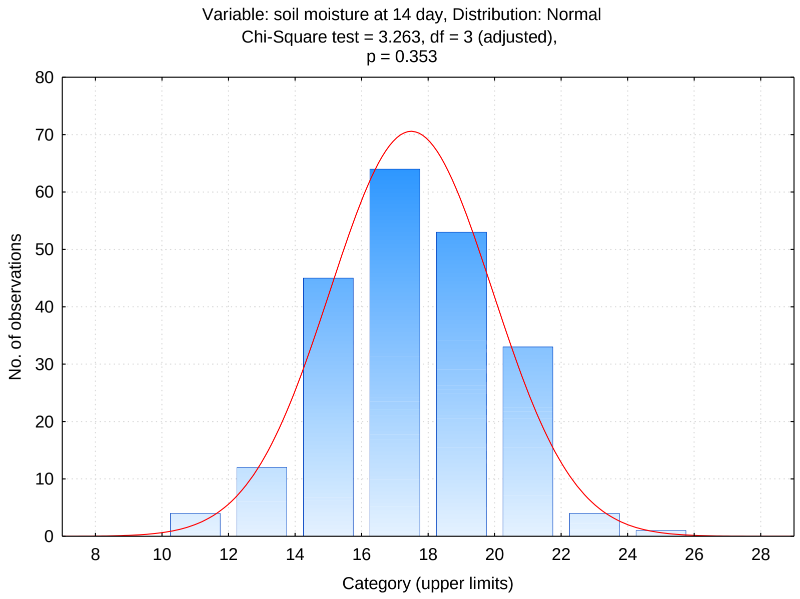

Figure S4. Distribution of soil moisture data recorded on the 14th day. .

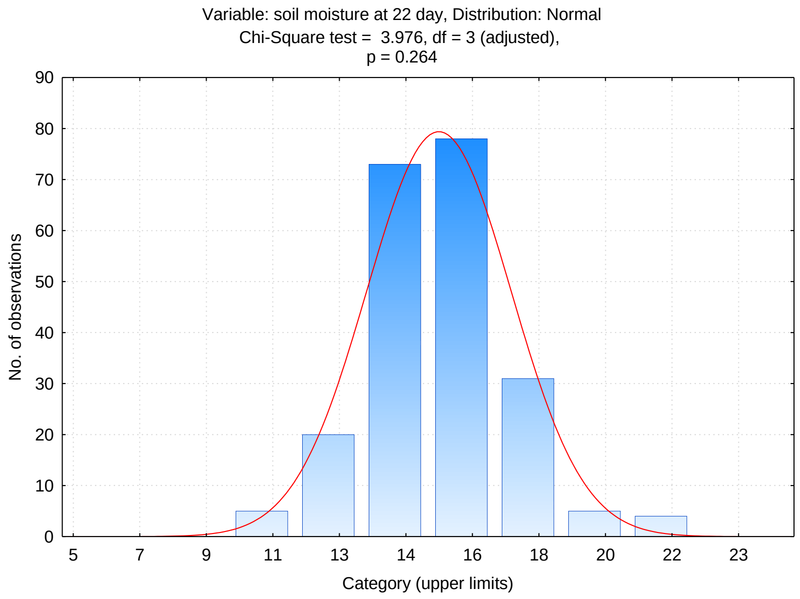

Figure S5. Distribution of soil moisture data recorded on the 22nd day. .

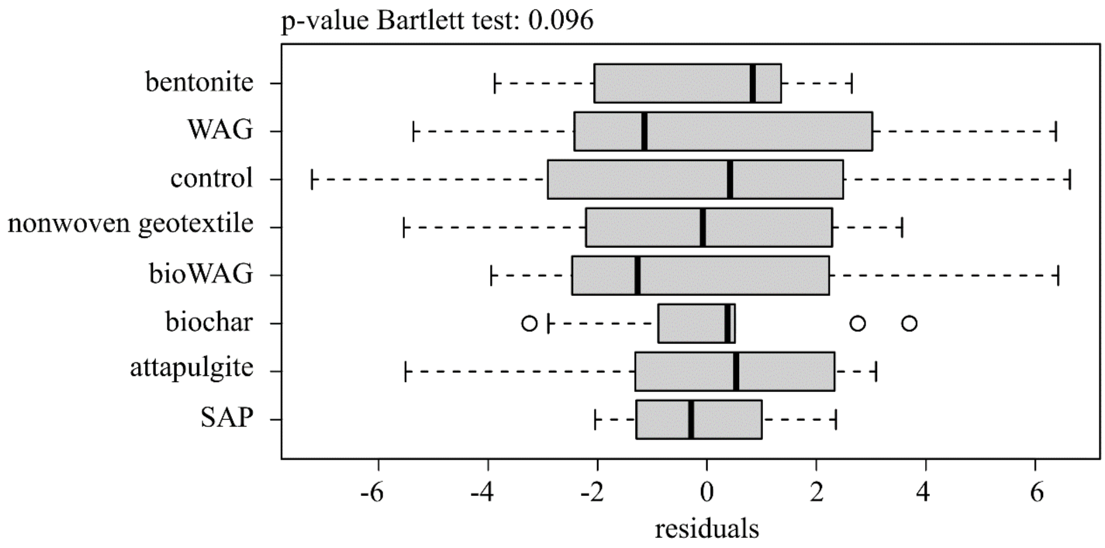

Figure S6. The homoscedasticity of residuals grouped by soil amendments on the 4th day.

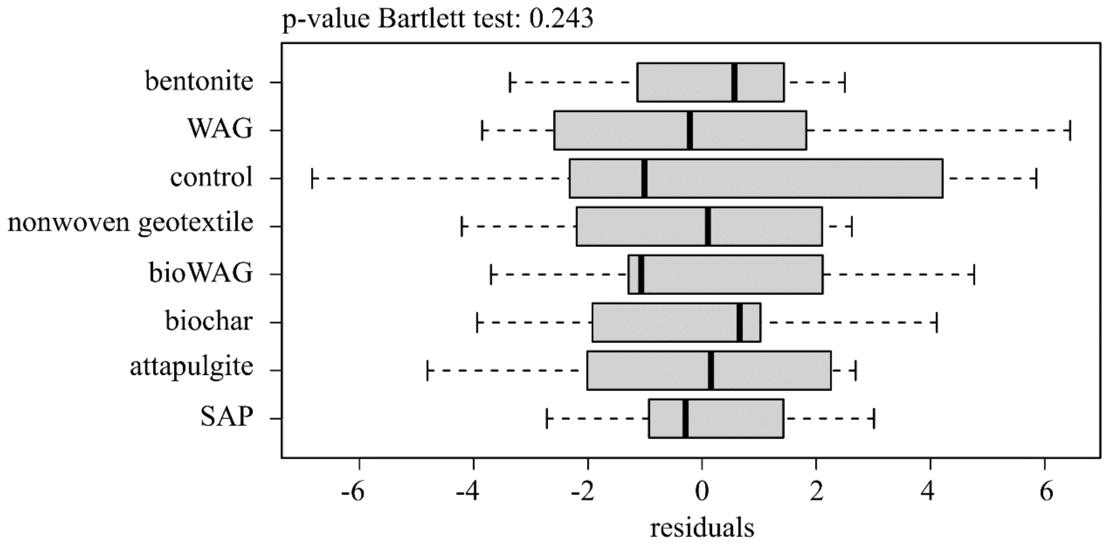

Figure S7. The homoscedasticity of residuals grouped by soil amendments on the 7th day.

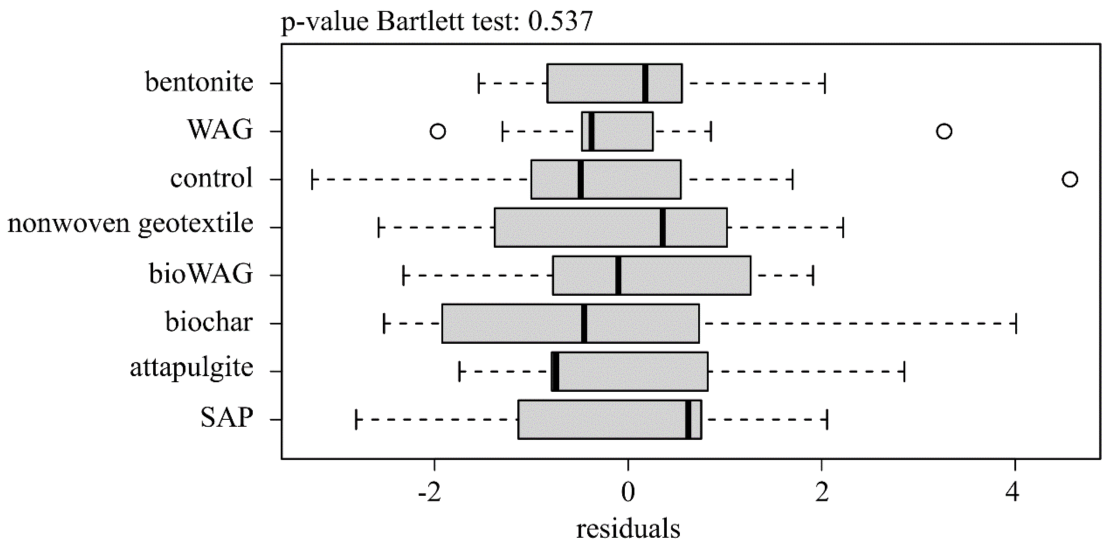

Figure S8. The homoscedasticity of residuals grouped by soil amendments on the 14th day.

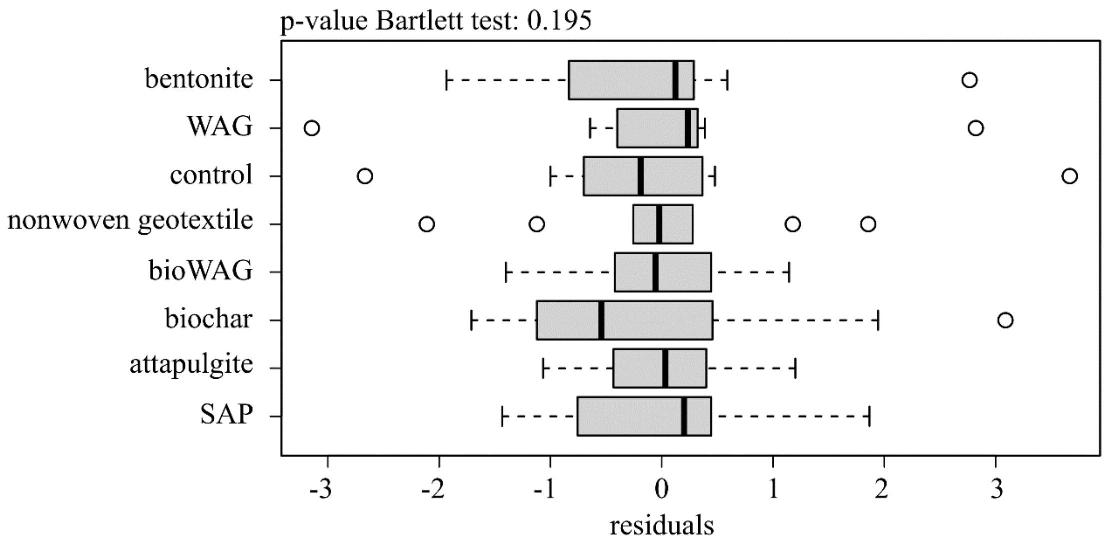

Figure S9. The homoscedasticity of residuals grouped by soil amendments on the 22nd day.

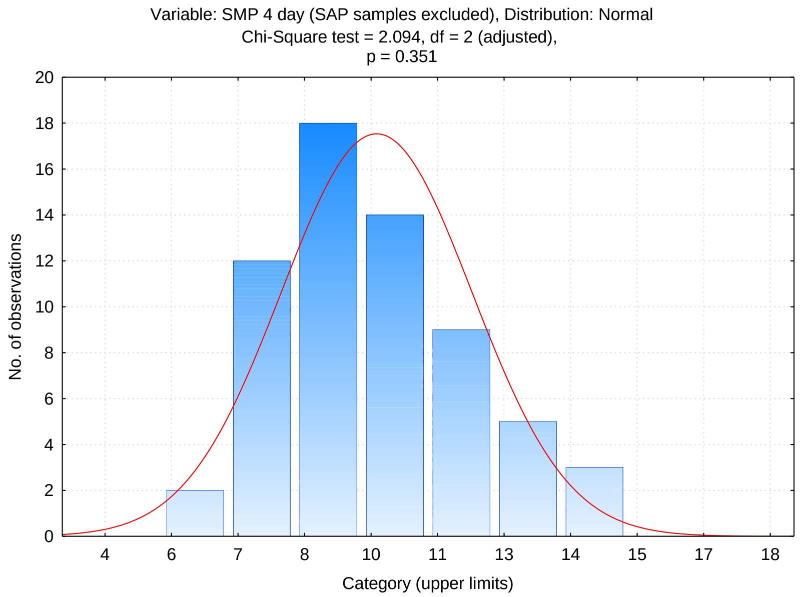

Figure S10. Distribution of soil matric potential (SMP) data recorded on the 4th day.

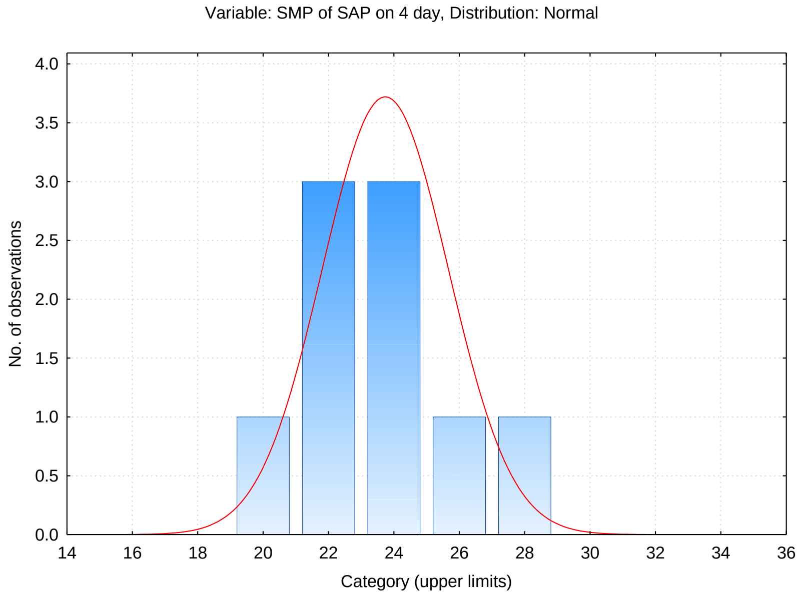

Figure S11. Distribution of soil matric potential (SMP) data of SAP samples recorded on the 4th day.

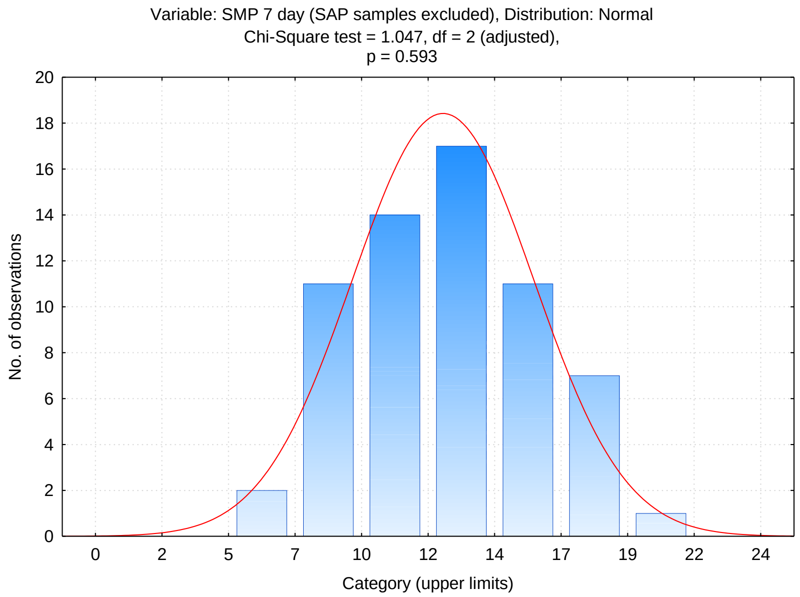

Figure S12. Distribution of soil matric potential (SMP) data recorded on the 7th day.

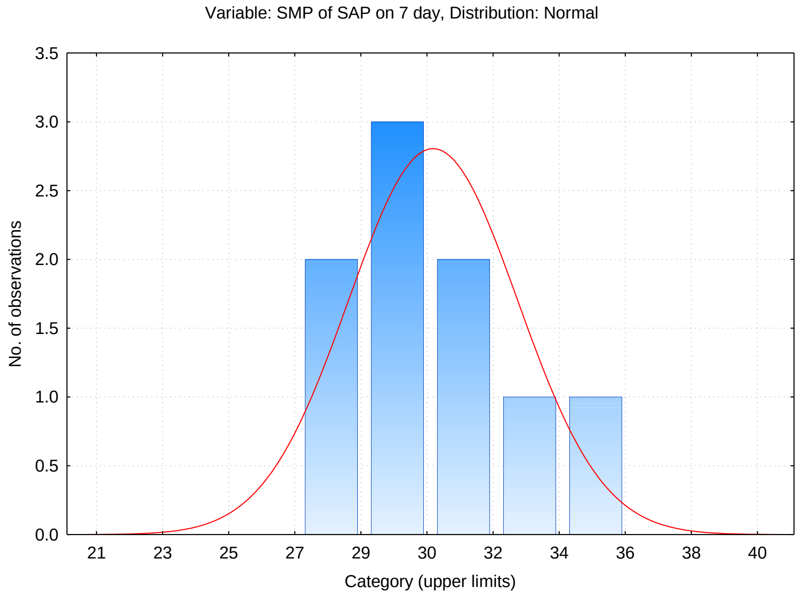

Figure S13. Distribution of soil matric potential (SMP) data of SAP samples recorded on the 4th day.

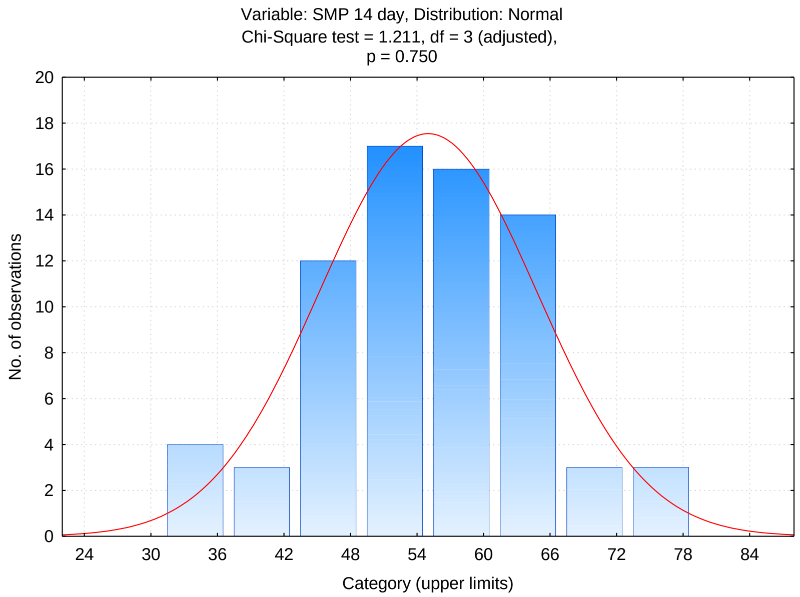

Figure S14. Distribution of soil matric potential (SMP) data recorded on the 14th day.

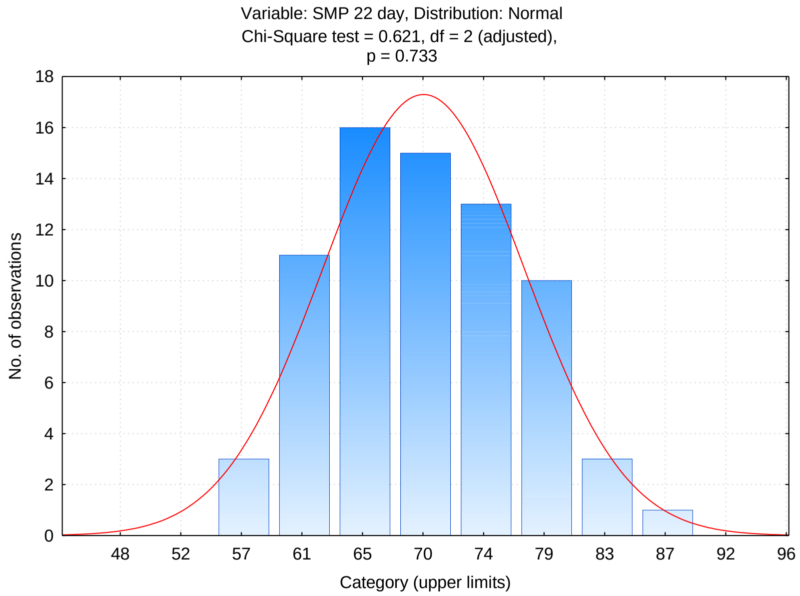

Figure S15. Distribution of soil matric potential (SMP) data recorded on the 22nd day.

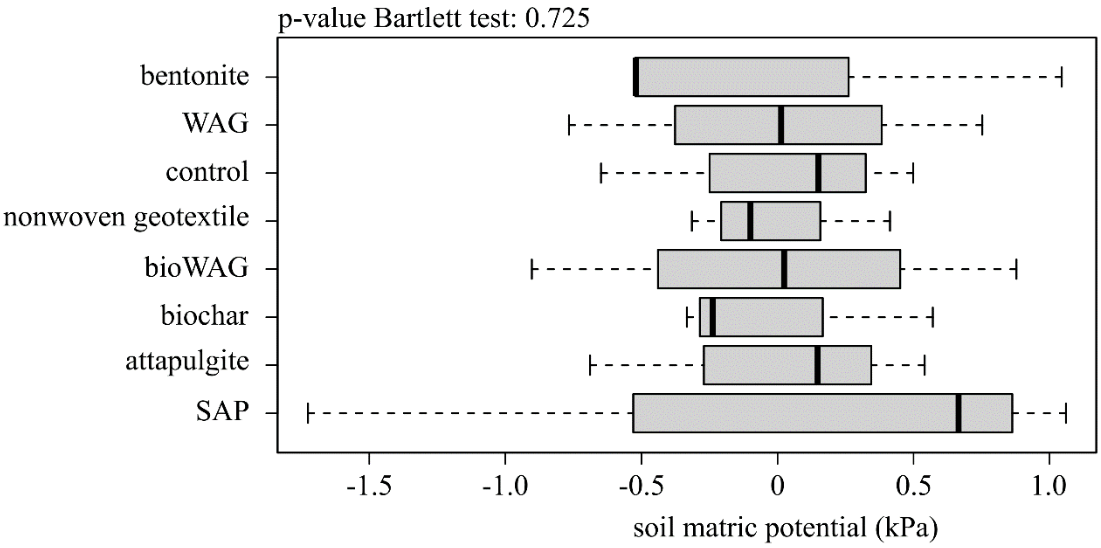

Figure S16. The homoscedasticity of residuals grouped by soil amendments on the 4th day.

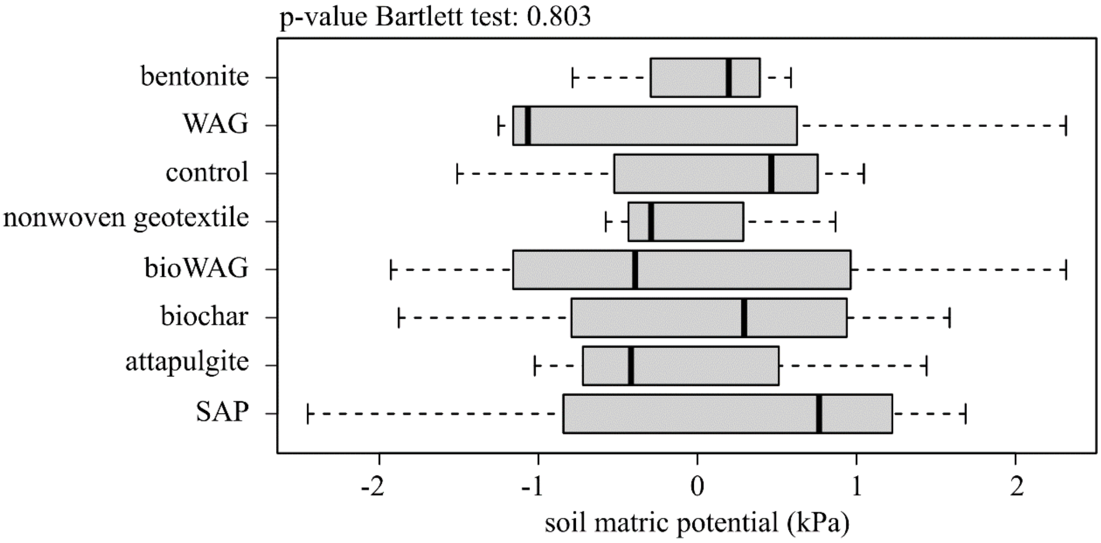

Figure S17. The homoscedasticity of residuals grouped by soil amendments on the 7th day.

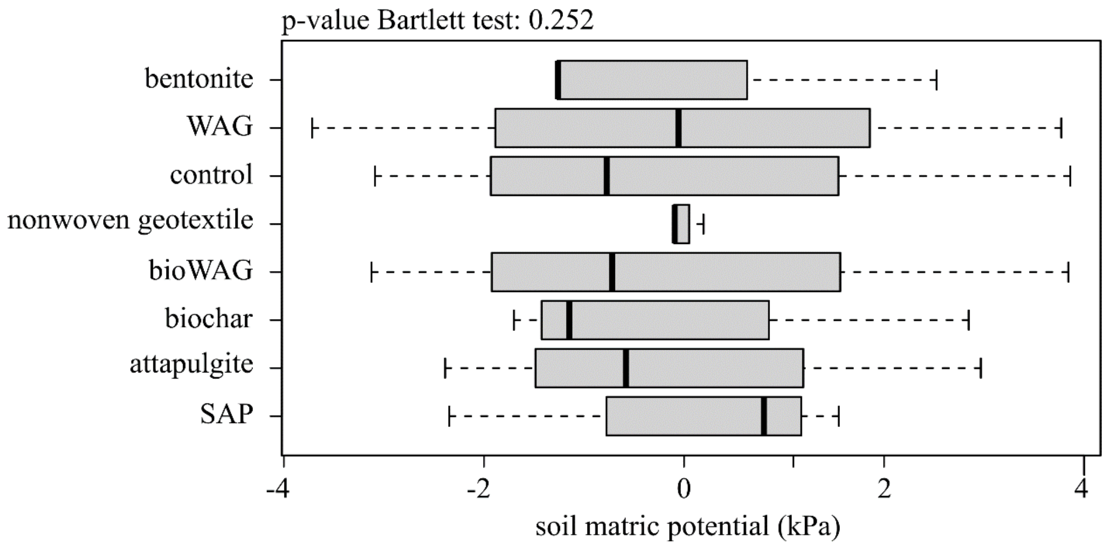

Figure S18. The homoscedasticity of residuals grouped by soil amendments on the 14th day.

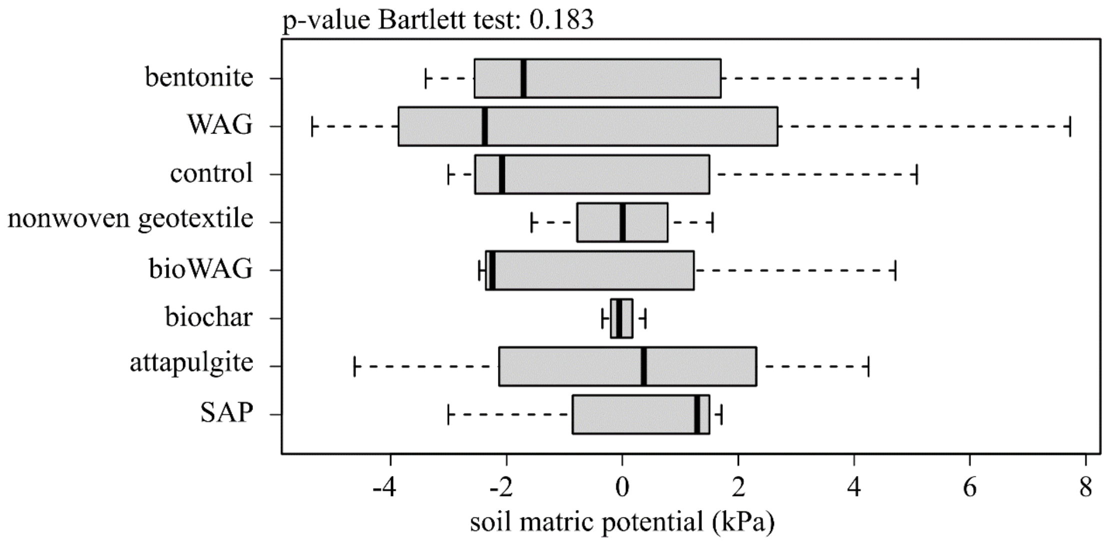

Figure S19. The homoscedasticity of residuals grouped by soil amendments on the 22th day.

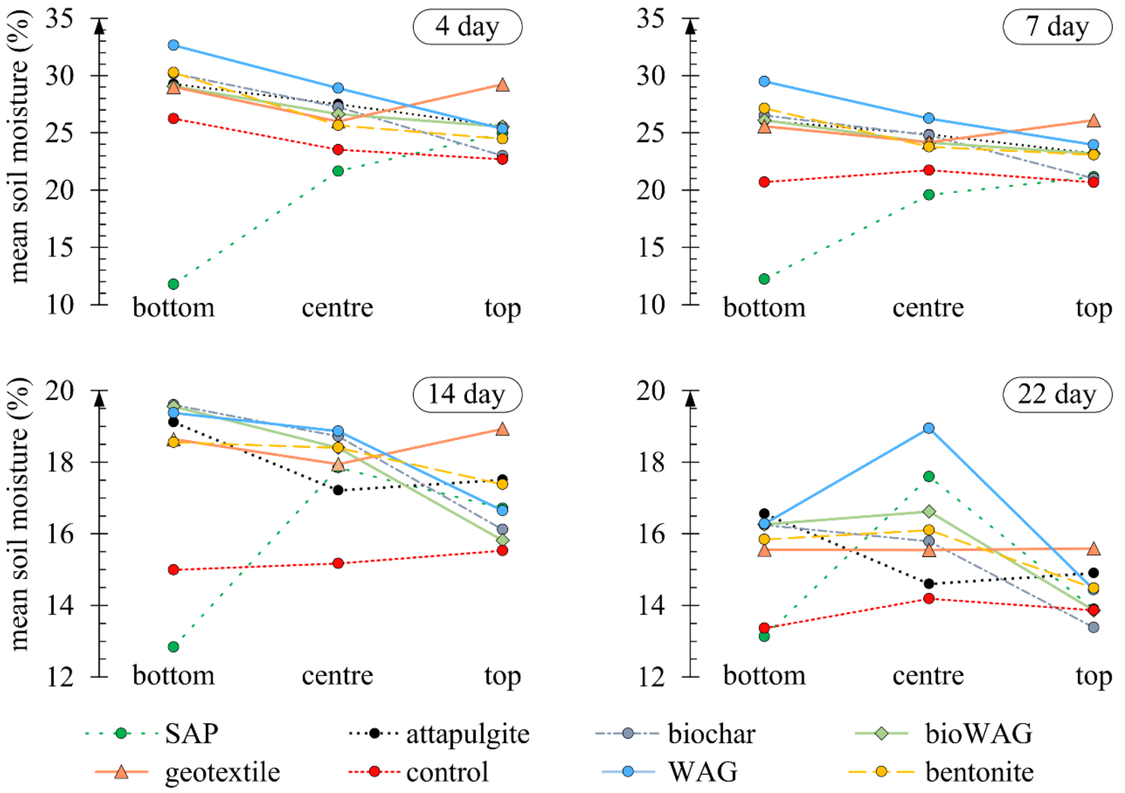

Figure S20. Mean soil moisture of pot samples at three depths of measurement (bottom, centre, top). Observations were taken during 22-day long drying cycles. Mean values were based on values of 3 sample replications and three repetitions of the experiment.
